# Identification of Genomic Instability-Associated LncRNAs as Potential Therapeutic Targets in Lung Adenocarcinoma

**DOI:** 10.3390/cancers17060996

**Published:** 2025-03-15

**Authors:** Vanessa G. P. Souza, Katya H. Benard, Greg L. Stewart, Katey S. S. Enfield, Wan L. Lam

**Affiliations:** 1 British Columbia Cancer Research Institute, Vancouver, BC V5Z 1L3, Canadawanlam@bccrc.ca (W.L.L.); 2 Interdisciplinary Oncology Program, University of British Columbia, Vancouver, BC V6T 1Z4, Canada; 3 Department of Pathology and Laboratory Medicine, University of British Columbia, Vancouver, BC V6T 1Z7, Canada

**Keywords:** genomic instability, homologous recombination deficiency (HRD), long non-coding RNAs (lncRNAs), non-small cell lung cancer (NSCLC)

## Abstract

Background/Objectives: Non-small cell lung cancer (NSCLC) is the most common type of cancer, with lung adenocarcinoma (LUAD) as the predominant subtype. Despite advancements in targeted therapies, many NSCLC patients still experience poor outcomes due to treatment resistance and disease progression. Genomic instability (GI), a hallmark of cancer, defined as the increased tendency of DNA mutations and alterations, is closely linked to cancer initiation, progression, and resistance to therapy. Emerging evidence suggests that long non-coding RNAs (lncRNAs)—molecules longer than 200 nucleotides that do not encode proteins but regulate gene expression—play critical roles in cancer biology and are associated with GI. However, the relationship between GI and lncRNA expression in LUAD remains poorly understood. Methods: In this study, we analyzed the transcript profiles of lncRNAs and mRNAs from LUAD samples in The Cancer Genome Atlas (TCGA) database and classified them based on their Homologous Recombination Deficiency (HRD) score. The HRD score is an unweighted sum of three independent DNA-based measures of genomic instability: loss of heterozygosity, telomeric allelic imbalance, and large-scale transitions. We then performed a differential gene expression analysis to identify lncRNAs and mRNAs that were either upregulated or downregulated in samples with high HRD scores compared to those with low HRD scores. Following this, we conducted a correlation analysis to assess the significance of the association between HRD scores and the expression of both lncRNAs and mRNAs. Results: We identified 30 differentially expressed lncRNAs and 200 mRNAs associated with genomic instability. Using an RNA interactome database from sequencing experiments, we found evidence of interactions between GI-associated lncRNAs (GI-lncRNAs) and GI-associated mRNAs (GI-mRNAs). Further investigation showed that some GI-lncRNAs play regulatory and functional roles in LUAD and other diseases. We also found that GI-lncRNAs have potential as prognostic biomarkers, particularly when integrated with HRD stratification. The expression of specific GI-lncRNAs was associated with primary therapy response and immune infiltration in LUAD. Additionally, we identified existing drugs that could modulate GI-lncRNAs, offering potential therapeutic strategies to address GI in LUAD. Conclusions: Our findings suggest that GI-associated lncRNAs could serve as valuable biomarkers for LUAD prognosis and therapeutic response. Furthermore, modulating these lncRNAs presents potential treatment avenues to address genomic instability in LUAD.

## Simple Summary

Non-small cell lung cancer (NSCLC) is the most common type of lung cancer, with lung adenocarcinoma (LUAD) being its predominant subtype. Despite advancements in treatment, LUAD outcomes remain poor due to treatment resistance, which is in part driven by genomic instability (GI)—an increased tendency for DNA mutations and other genetic changes that occur during cell division. Using LUAD samples from The Cancer Genome Atlas (TCGA) database, we classified cases by Homologous Recombination Deficiency (HRD) score—a measure of GI—and identified 30 long non-coding RNAs (lncRNAs) and 200 messenger RNAs (mRNAs) associated with GI. We found that GI-associated lncRNAs (GI-lncRNAs) could serve as prognostic biomarkers, particularly when combined with HRD score. These lncRNAs were also linked to therapy response and immune infiltration in high-GI samples. Additionally, we identified existing drugs that could interact with specific GI-lncRNAs, offering opportunities for drug repurposing in LUAD or for experimental research into their mechanistic roles in tumor progression.

## 1. Introduction

Lung cancer is the leading cause of cancer-related deaths worldwide, representing roughly 18% of all cancer-related deaths [1]. Non-small cell lung cancer (NSCLC) is the predominant subtype, accounting for approximately 87% of cases [2]. Due to the lack of symptoms, a substantial proportion of NSCLC patients are diagnosed at locally advanced or metastatic stages, with frequent spread to organs such as the liver, brain, and bones, leading to poor survival outcomes [3,4]. The five-year survival rate for NSCLC depends on the stage at diagnosis, varying from 65% when diagnosed at an early stage to 9% in advanced stages [5].

Targeted therapies have significantly improved outcomes for NSCLC patients, making broad-panel molecular testing essential for identifying actionable genetic alterations. Tyrosine kinase inhibitors (TKIs) that target genetic mutations in *EGFR*, *ALK*, *ROS1*, *RET*, *BRAF*, *MET* exon 14, and *NTRK* have been approved for different subtypes of NSCLC [6]. However, only a modest fraction of patients have these mutations, and for most patients without driver mutations, chemoimmunotherapy is the preferred first-line treatment [7]. Combining chemotherapy with immunotherapy typically results in an increased median progression-free survival (PFS) and median overall survival (OS) for both squamous and non-squamous NSCLC in advanced stages [8,9]. Despite initial benefits, most patients experience disease progression within the first year of treatment. Consequently, second-line treatment with docetaxel monotherapy remains the standard of care for most patients with advanced NSCLC without actionable genomic alterations, despite its modest efficacy and notable toxicity [7,10].

A central challenge in NSCLC treatment is therapy resistance, with systemic progression often arising due to drug resistance in tumor cells [11]. Although the molecular basis of drug resistance is complex, genomic instability (GI)—a key driver of tumor evolution and intratumor heterogeneity—is increasingly recognized as a major contributor [12,13,14]. Through GI, tumor cells can adopt diverse phenotypes, enhancing their survival under the selective pressures of treatment [14,15]. This adaptability highlights GI’s role in cancer progression and poor therapeutic outcomes, fueling a growing interest in targeting GI and aneuploidy as therapeutic strategies [16].

Lung adenocarcinoma (LUAD) exhibits higher GI scores than many other tumor types [17], with chromosomal instability linked to poorer prognosis and metastasis rates [18]. In LUAD, GI-related genes such as *PPAT* and *PAICS*, as well as immune-related pathways like *MEC2C* and *CCL14-CCR1*, have been proposed as potential therapeutic targets [19,20].

While studies have shown that GI may serve as a prognostic marker in cancers like cervical and breast cancer [21,22] and emerging evidence links GI to lung cancer tumorigenesis [23,24], the prognostic significance of GI in LUAD remains inconsistent across different studies and subgroups. This inconsistency suggests that the prognostic role of GI in LUAD may vary based on factors such as genomic alterations, clinical characteristics, and treatment regimens [25,26,27,28].

GI in malignant tumors can be broadly classified into four types: chromosomal instability (CIN), intrachromosomal instability, microsatellite instability, and epigenetic instability [29]. CIN is the most common form of GI observed in over 90% of solid tumors and many blood cancers [30,31,32]. Although GI is a recognized cancer hallmark [33], it remains unclear whether it is primarily a cause or consequence of cancer progression, and its underlying genetic vulnerabilities are not fully understood [29].

Recently, studies have shown that GI can be evaluated using the Homologous Recombination Deficiency (HRD) score [25,34,35,36]. The HRD score is used to assess the dysfunction in the homologous recombination repair (HRR) pathway at the cellular level [37]. HRR is responsible for precise DNA damage repair, especially double-strand break (DSB) repair [26,37,38]. When HRR is impaired, as in some cancers, cells accumulate genetic alterations such as abnormal copy numbers and chromosomal cross-linking, leading to both genomic instability and CIN [37]. By calculating a combined score of events such as loss of heterozygosity (LOH), large-scale state transitions (LSTs), and telomere allele imbalance (TAI), the HRD score provides a quantifiable assessment of genomic scarring, offering a comprehensive score for the evaluation of GI in cancer [34,35,37]. The concept of HRD scoring was first introduced and validated in cancer genomics studies, such as those by Abkevich et al. (2012) [39] and Timms et al. (2014) [34], and HRD scoring was further validated as a predictor of therapy response, particularly for DNA-damaging treatments like platinum-based chemotherapy and PARP inhibitors [40,41,42].

Long non-coding RNAs (lncRNAs) are transcripts longer than 200 nucleotides that lack protein-coding potential but play essential roles in maintaining normal cellular functions and homeostasis. They regulate gene expression at multiple levels, including epigenetic modification, transcriptional regulation, and post-transcriptional control [43,44]. The roles of lncRNAs in cancer biology are increasingly recognized, particularly in processes like cell proliferation, differentiation, and genomic stability [45,46]. Notably, certain lncRNAs, including *NORAD*, have been shown to play direct roles in chromosomal stability [47,48]. However, only a few genomic instability-associated lncRNAs (GI-lncRNAs) have been identified, and their involvement in LUAD and their association with HRD scores have not been fully explored [47].

Recent studies have shown that genomic instability-related lncRNA signatures (GILncSig) could serve as independent predictors of survival in LUAD [49,50]. For instance, a model incorporating five GI-associated lncRNAs, developed using data from The Cancer Genome Atlas (TCGA), was identified as an independent predictor of OS in LUAD patients [50]. These findings underscore the promising role of GI-associated lncRNAs as biomarkers for prognosis.

Beyond their biomarker potential, lncRNAs are promising pharmacological targets [51]. However, developing new drugs is a lengthy process, often requiring years to complete all design and approval phases. Drug repurposing has been utilized to identify new therapeutic roles for existing drugs [52]. LncRNAs have emerged as important players in drug repurposing efforts. For example, one study identified drugs that could modulate lncRNAs involved in the cross-talk between diabetes mellitus and Alzheimer’s disease, revealing new therapeutic possibilities [53]. Another analysis identified over 27,000 lncRNA–drug interactions with 265 chemotherapy drugs, uncovering “multi-drug resistant” lncRNAs linked to resistance against more than 100 chemotherapy agents, suggesting significant roles in drug metabolism and disposition [54]. Additionally, LncTx—a network-based tool—identifies potential drugs that act on survival-related lncRNAs in lung cancer [55]. Given this, identifying GI-lncRNAs in LUAD could provide new insights into the molecular underpinnings of this cancer and reveal potential therapeutic targets.

Therefore, the rationale for this study is to investigate the role of GI-lncRNAs in LUAD, with the aim of identifying potential biomarkers for prognosis and novel therapeutic targets to improve clinical outcomes. We explore the potential mechanisms regulating these lncRNAs using in silico analysis. Additionally, by examining the relationship between HRD scores and the mutational landscape, we analyze how alterations in key driver genes may influence genomic instability and contribute to tumorigenesis and LUAD progression. We conclude that GI-lncRNAs could serve as potential therapeutic targets and offer new opportunities for drug repurposing and precision treatment strategies for LUAD.

## 2. Materials and Methods

**Figure 1:**
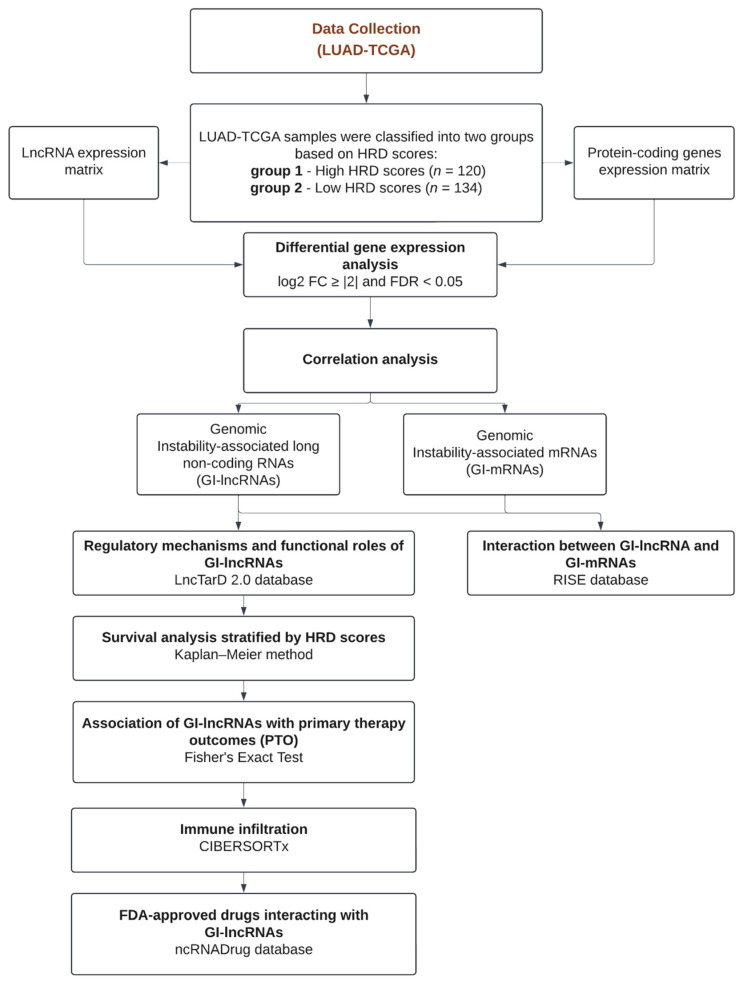
Homologous Recombination Deficiency (HRD) scores for LUAD-TCGA samples were obtained from a previous study, which calculated HRD scores based on three indicators: loss of heterozygosity (LOH), telomeric allelic imbalance (NtAI), and large-scale state transitions (LSTs) [37]. Using these HRD scores, samples were classified into two groups: high HRD scores and low HRD scores. RNA sequencing data and corresponding clinicopathological features for these selected samples were retrieved from the TCGA database (details provided in the methodology section). LncRNA and mRNA expression profiles were extracted from TCGA gene expression quantification files (counts), and differential expression analysis was performed to identify lncRNAs and mRNAs up- or downregulated in the high-HRD group. Thresholds for differential expression were set to log2 fold change ≥ |2| and FDR < 0.05. A Spearman correlation test was then conducted to identify differentially expressed lncRNAs and mRNAs that also correlated with HRD scores. LncRNAs meeting both differential expression (log2 fold change ≥ |2|, FDR < 0.05) and correlation (Spearman coefficient ≥ |0.2|, *p*-value < 0.05) criteria were classified as genomic instability-associated lncRNAs (GI-lncRNAs). Similarly, mRNAs meeting a Spearman coefficient ≥ |0.1| with a *p*-value < 0.05 were classified as genomic instability-associated mRNAs (GI-mRNAs). To explore the regulatory roles and functions of GI-lncRNAs in human diseases, we used the LncTarD 2.0 database [56], and RNA–RNA interactions between GI-lncRNAs and GI-mRNAs were examined using the RISE database [57]. The clinical significance of GI-lncRNAs as prognostic biomarkers in LUAD was assessed through survival analysis, stratifying LUAD patients by HRD scores. Additionally, we investigated the potential relevance of GI-lncRNAs in predicting therapy response in patients with high HRD scores. Differences in immune cell infiltration between patients with high and low HRD scores were analyzed using the CIBERSORTx algorithm [58]. Finally, potential drug candidates targeting GI-lncRNAs were identified using the ncRNADrug database [59].

A schematic overview of the methodology is provided in Figure 1.

### 2.1. Data Collection and HRD Scores

Homologous Recombination Deficiency (HRD) scores for LUAD-TCGA samples were obtained from a previous study [37], which utilized three key indicators—loss of heterozygosity (LOH), telomeric allelic imbalance (NtAI), and large-scale state transitions (LSTs)—to determine the HRD status of each sample. Based on these HRD scores, we classified LUAD-TCGA samples into quartiles (Q1–Q4) and then selected the top 25% of samples with the highest HRD scores (Q4: high HRD scores) and the bottom 25% with the lowest HRD scores (Q1: low HRD scores), following the methodology outlined in a previous study [60] (Supplementary Table S1). Using the sample IDs for LUAD-TCGA samples provided in this study, we downloaded the RNA sequencing transcriptome data and the corresponding clinicopathological features of LUAD patients from the TCGA database [61]. Comparative statistical analysis was performed using the Chi-Square test to assess the association between clinicopathological characteristics and HRD scores. The clinicopathological characteristics of the patients in this study are presented in Table 1. Detailed clinical information for each sample can be found in Supplementary Table S2.

### 2.2. Analysis of Gene Mutational Status and HRD Scores in Lung Adenocarcinoma

The mutational status of key driver genes in LUAD (*TP53*, *EGFR*, *KRAS*, *PIK3CA*, and *CDKN2A*) was retrieved from the UCSC Xena database [62] using the GDC TCGA Lung Adenocarcinoma (LUAD) dataset. Somatic mutation data for each selected gene were downloaded and imported into R software (v.4.4.1) [63]. In the R environment, we filtered the GDC TCGA LUAD dataset to include only samples with high and low HRD scores. To explore the relationship between mutational status and HRD scores (high vs. low), a Chi-Square test was performed using the chisq.test function from the stats package (v.4.4.1) available in R.

### 2.3. Identification of Genomic Instability-Associated Long Non-Coding RNAs (GI-lncRNAs) and mRNAs (GI-mRNAs), and Differentially Expressed miRNAs (DE-miRNAs) 

Transcriptome data for RNA, miRNA, and clinicopathological features were obtained from the TCGA database and analyzed in R software (v.4.4.1) [61,64]. Seven transcriptomic samples with incomplete clinical data were excluded to ensure that only fully annotated samples were included in the analysis, maintaining the integrity of the dataset. LncRNA and mRNA expression profiles were extracted from the gene expression quantification files (counts), which contain raw count data for each gene in the dataset. Genes identified as “lncRNA” in the count file were categorized as lncRNAs (*n* = 16,901), and genes labeled as “protein_coding” were categorized as mRNAs (*n* = 19,962). MiRNA expression levels were extracted from the corresponding miRNA quantification files (mirnas.quantification.txt). To explore sources of variation in the dataset, we performed Principal Component Analysis (PCA), a widely used dimensionality reduction and visualization method for high-dimensional data. This analysis was conducted using the prcomp function from the stats package (v.4.4.1) in R. The expression matrix was used as input to assess potential batch-related variation in the data.

Subsequently, we employed the EdgeR package (v.3.19) [65] for differential expression analysis. EdgeR is a widely used tool for RNA-seq data due to its robust normalization and statistical modeling capabilities. It employs the Trimmed Mean of M-values (TMM) method via the calcNormFactors() function, a robust approach for handling variations in library size, which is a common characteristic of RNA-seq data [66,67]. The filterByExpr function was used to exclude genes that were not expressed in sufficient samples to ensure statistical robustness as genes with low expression levels are more likely to produce unreliable results. For differential expression analysis of lncRNAs and mRNAs, we set thresholds of a log2 fold change ≥ |2| and an FDR < 0.05. For miRNAs, we did not consider log2 fold change and used only an FDR < 0.05 as the threshold.

In addition to differential expression, we performed a Spearman correlation test using the cor.test function from the stats package (v.4.4.1) available in R to assess the association between HRD scores and the expression of lncRNAs and mRNAs. The Spearman correlation was selected because it is a non-parametric test that does not assume a linear relationship, making it appropriate for capturing more complex associations between HRD scores and gene expression data. We set the threshold for correlation significance at a p-value < 0.05, with a minimum Spearman coefficient ≥ |0.2|, a threshold commonly used to highlight meaningful but potentially weak correlations in large genomic datasets.

LncRNAs were classified as genomic instability-associated long non-coding RNAs (GI-lncRNAs) if they met both of the following criteria: (a) differential expression (log2 fold change ≥ |2| and an FDR < 0.05) and (b) Spearman coefficient ≥ |0.2| with *p*-value < 0.05. Similarly, mRNAs that met the criteria of (a) differential expression (log2 fold change ≥ |2| and an FDR < 0.05) and (b) Spearman coefficient ≥ |0.1| with *p*-value < 0.05 were classified as genomic instability-associated mRNAs (GI-mRNAs). The genomic locations for both GI-lncRNAs and GI-mRNAs were identified using GeneCards^®^: The Human Gene Database [68], based on the latest assembly (GRCh38/hg38).

### 2.4. Pathway Enrichment Analysis

For GI-associated lncRNAs, Kyoto Encyclopedia of Genes and Genomes (KEGG) pathway enrichment analysis was conducted using the LncRNAs2Pathways tool [69], with an FDR < 0.05 considered significant. For GI-mRNAs, Kyoto Encyclopedia of Genes and Genomes (KEGG) and Gene Ontology (biological process) enrichment analyses were performed using Enrichr [70,71,72]. We set *p* < 0.05 as the threshold for statistical significance.

### 2.5. Interactions Between GI-lncRNA and GI-mRNAs, Along with the Regulatory Mechanisms and Functional Roles of GI-lncRNAs

We aimed to select the most reliable interactions involving GI-lncRNAs and GI-mRNAs. To achieve this, we downloaded RNA–RNA interactions from the RISE database [57]. This comprehensive database of RNA interactomes from sequencing experiments includes 10,941 lncRNA–mRNA interactions. RISE provides an extensive collection of RNA–RNA interactions (RRIs) identified in humans, mice, and yeast, along with detailed molecular annotations for each RRI. The RRIs were curated from transcriptome-wide and targeted sequencing studies, as well as other public databases and datasets [57]. We downloaded the complete RNA–RNA interactome for humans and imported the dataset into R for further analysis. In the R environment, we selected only interactions involving the GI-lncRNAs of interest and GI-mRNAs. Subsequently, we conducted a Spearman correlation analysis between the GI-lncRNAs and GI-mRNAs to identify only those with interactions in our cohort (*p* < 0.05). The lncRNA–mRNA interaction network in our model was constructed based on these interactions using Cytoscape software (v.3.10.1) [73]. Additionally, the LncTarD 2.0 database was used to explore the associated functions and regulatory mechanisms of GI-lncRNAs in human diseases, including LUAD [56].

### 2.6. Patient Survival Analysis

Recurrence, death, and progression-free survival data were downloaded from the TCGA database [61] and used to assess overall survival (OS) and progression-free survival (PFS). We normalized gene expression using the z-score formula, i.e., z = (x − x^−^)/s, where x represents the gene expression value, x^−^ is the mean of gene expression values, and s is the standard deviation. Associations between gene expression and OS were investigated following Sha’s method [74]. Briefly, patients were categorized based on gene expression levels into ’high’ (above the median) and ’low’ (below the median) groups. The prognostic significance of these classifications was assessed through survival analysis. OS and PFS were estimated using the Kaplan–Meier method by making comparisons between groups using the log-rank test. The analysis utilized the survival (v.3.7-0) [75] and survminer (v.0.4.9) [76] packages in R. Statistical significance was defined as a *p* < 0.05.

### 2.7. Patient Treatment Response

Data on primary therapy outcome success and follow-up treatment success for LUAD-TCGA samples were downloaded from the UCSC Xena database [62] using the GDC TCGA Lung Adenocarcinoma (LUAD) dataset. Gene expression was normalized using the z-score, as previously described. Patients were categorized based on gene expression levels into ’high’ (above the median) and ’low’ (below the median) groups. We then performed a Fisher’s Exact Test to assess the relationship between gene expression categories (“high” vs. “low”) and therapy outcome success (“complete remission/response”, “stable disease”, and “progressive disease”) for each group (high HRD scores and low HRD scores). The Fisher’s Exact Test was performed using the fisher.test function from the stats package (v.4.4.1). Statistical significance was defined as an adjusted *p*-value < 0.05.

### 2.8. Immune-Related Analysis

To assess the immune composition of the samples, we utilized the CIBERSORTx tool [58]. This tool incorporates the LM22 reference file, which is a signature matrix containing 547 genes that differentiate 22 distinct human hematopoietic cell populations, including 7 T cell subtypes, B cells, plasma cells, and NK cells and various myeloid subsets. Prior to analysis in CIBERSORTx, raw RNA-seq counts were converted to transcripts per million (TPM) using the countToTpm() function from the R package GeoTcgaData (v.2.4.0) [77]. Immune cell profiling was conducted with CIBERSORTx, using 1000 permutations for enhanced statistical reliability, while quantile normalization was disabled. The resulting output files were downloaded in tab-delimited format and subsequently imported into R (v.4.4.1), which was used to identify differences in immune composition between high and low HRD scores. The normality test was performed using the shapiro.test function. Differences were considered significant when *p* < 0.05 based on the Wilcoxon–Mann–Whitney test. Then, we explored the association between the populations of immune cells and the expression of GI-lncRNAs; for this, the Spearman correlation analysis was performed using the cor.test function of the R stats package (v.4.4.1) [63]. Values were considered significant when *p* < 0.05. Both raw and TPM-normalized counts were used in the correlation analysis to ensure that the correlations remained consistent across both methods. The plots were generated using the package ggplot2 (v.3.5.1) [78].

### 2.9. Identification of Candidate Drugs Targeting GI-lncRNAs

To identify potential drug candidates targeting GI-lncRNAs, we utilized the ncRNADrug database, a comprehensive resource for drug and ncRNA associations. This database includes both experimentally validated and computationally predicted ncRNAs linked to drug resistance, as well as ncRNAs targeted by specific drugs [79].

## 3. Results

### 3.1. Characterization of HRD Scores and Their Association with Clinicopathological Features in LUAD-TCGA Samples

HRD scores for LUAD-TCGA samples were obtained as outlined in the methodology (Figure 1). Based on these HRD scores, we classified LUAD-TCGA samples into quartiles (Q1–Q4) (Supplementary Table S1). We then selected the top 25% of samples with the highest HRD scores (Q4: high HRD scores, *n* = 122) and the bottom 25% with the lowest HRD scores (Q1: low HRD scores, *n* = 134), following the methodology outlined in a previous study [60]. The samples TCGA-50-5066-02 and TCGA-50-5946-02 were excluded from our subsequent analysis due to their classification as recurrent solid tumors. Research has shown that LUAD recurrent tumors have a higher mutation burden than non-recurrent tumors [80]. Following this removal, we retained 120 samples with high HRD scores and 134 samples with low HRD scores.

**Figure 2:**
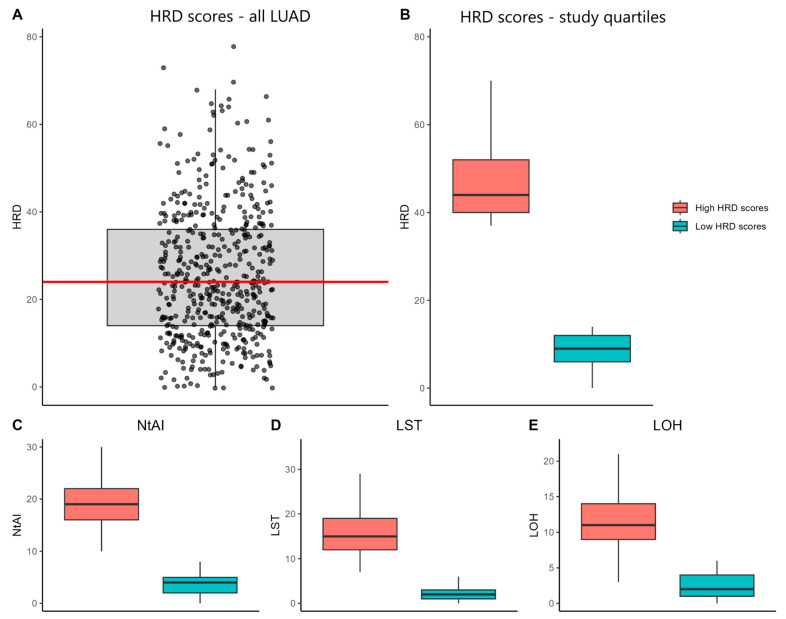
(**A**) Distribution of Homologous Recombination Deficiency (HRD) scores across all LUAD samples obtained from Shi et al., 2023 [37], with the median HRD score highlighted in red. (**B**) Distribution of HRD scores for the top 25% of samples classified as high HRD scores compared to the bottom 25% classified as low HRD scores used in this study. (**C**) Distribution of telomeric allelic imbalance (NtAI) scores among samples categorized by high and low HRD scores. (**D**) Distribution of large-scale state transition (LST) scores among samples categorized by high and low HRD scores. (**E**) Distribution of loss of heterozygosity (LOH) scores among samples categorized by high and low HRD scores. The HRD scores and NtAI, LST, and LOH values were obtained from Shi et al. (2023) [37]. In all plots, boxes represent the standard deviation, with whiskers extending to the 95th percentile.

The distribution of HRD scores across LUAD samples is displayed in Figure 2A. Based on these scores, samples were categorized into two groups: high HRD (*n* = 120) and low HRD (*n* = 134). The median HRD scores were 44 for the high-HRD group and 9 for the low-HRD group (Figure 2B). Median values for NtAI, LSTs, and LOH were markedly different between the groups, with the high-HRD group showing values of 19, 15, and 11, respectively, compared to 4, 2, and 2 in the low-HRD group (Figure 2C–E).

We analyzed the associations between HRD scores and clinicopathological features, including pathological stage, vital status, sex, race, tumor size (T), lymph node involvement (N), and metastasis (M). HRD scores were not significantly associated with most clinical characteristics, except for age (*p* = 0.001, Chi-Square test) (Table 1). Additionally, there were no differences in OS or PFS between patients with high or low HRD scores (Supplementary Figure S1). These results align with those of previous studies [25].

Given the reported association between GI/HRD scores and genomic alterations in NSCLC [25], we investigated the relationship between the mutational status of key driver genes in LUAD and HDR scores (Table 2). *TP53* somatic mutations were significantly more prevalent in the group with high HRD scores, with 80% of patients exhibiting *TP53* alterations, compared to only 13.43% in the group with low HRD scores (*p* = 0.0001). We found no significant associations with other assessed genes.

### 3.2. Identification of Genomic Instability-Associated Long Non-Coding RNAs (GI-lncRNAs) in Lung Adenocarcinoma

**Figure 3:**
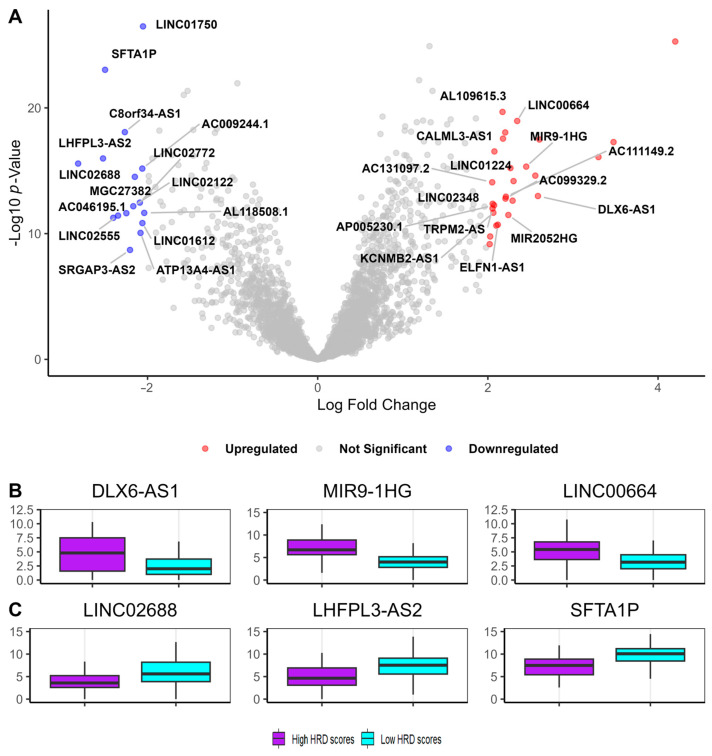
(**A**) Volcano plot showing upregulated and downregulated genes in samples with high HRD scores compared to those with low HRD scores. Red points represent upregulated long non-coding RNAs (lncRNAs), and blue points indicate downregulated lncRNAs, defined by a log2 fold change ≥ |2| and an FDR < 0.05. GI-associated lncRNAs—those that are both differentially expressed and significantly correlated with HRD scores (Spearman correlation)—are labeled. Gray points represent genes that are not significantly differentially expressed. (**B**) Box plots showing the expression levels (log2-transformed) of the top three upregulated GI-lncRNAs. (**C**) Box plots showing the expression levels (log2-transformed) of the top three downregulated GI-lncRNAs.

To identify GI-lncRNAs in LUAD, we first compared lncRNA expression between samples with high HRD scores and those with low HRD scores. This analysis revealed a total of 43 differentially expressed lncRNAs (DE-lncRNAs) (log2 FC ≥ |2| and FDR < 0.05). Among the DE-lncRNAs, 27 were upregulated and 16 were downregulated. The list of DE-lncRNAs is shown in Supplementary Table S3. Following the identification of DE-lncRNAs, we performed a correlation analysis to evaluate the significance and strength of the associations between HRD scores and DE-lncRNA expression levels. DE-lncRNAs with a Spearman correlation coefficient (|r| ≥ 0.2) and *p*-value < 0.05 were classified as genome instability-associated lncRNAs (GI-lncRNAs; Supplementary Table S4). This analysis identified 30 GI-lncRNAs, as illustrated in Figure 3A.

**Figure 4:**
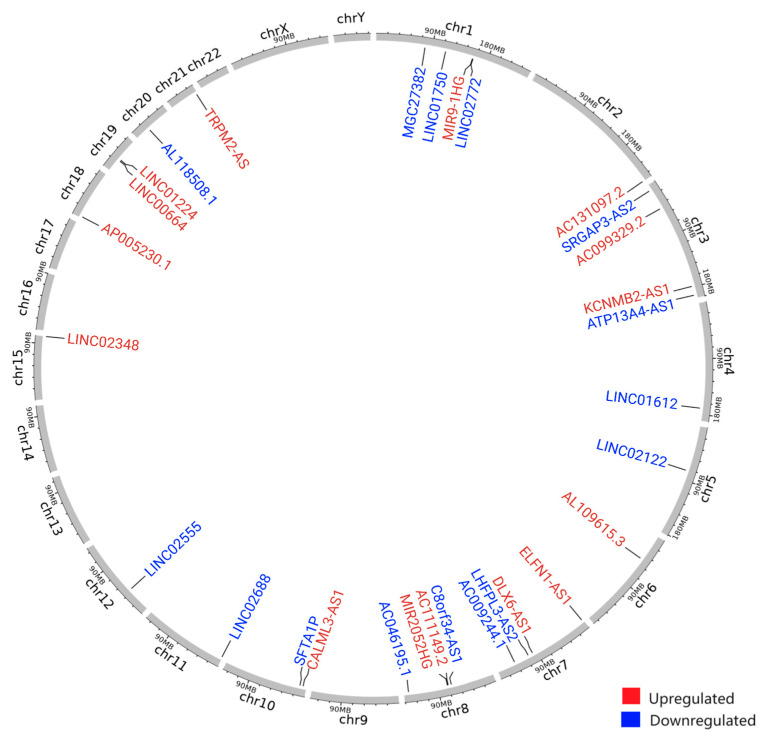
The circos plot illustrates the chromosomal distribution of the genomic instability-associated long non-coding RNAs (GI-lncRNAs). In this plot, each circle from the periphery to the center represents the following specifications: chromosomal location and the positions of the known genes in the genome. The upregulated GI-lncRNAs are colored in red, while the downregulated ones are colored in blue (with a log2 fold change ≥ |2| and an FDR < 0.05). This figure was created using shinyCircos-V2.0 [81].

Among the GI-lncRNAs, *DLX6-AS1* was the most upregulated (log2FC = 2.58) (Figure 3B), while *LINC02688* was the most downregulated (log2FC = −2.81) (Figure 3C). Using the LncRNAs2Pathways tool, we found that these GI-lncRNAs were significantly enriched in several KEGG pathways, including oxidative phosphorylation, chemokine signaling, and homologous recombination (FDR < 0.05) (Supplementary Table S5). Additionally, we explored the chromosomal locations of the GI-lncRNAs and found that they are widely distributed across the genome (Figure 4 and Supplementary Table S6).

### 3.3. Identification of Genomic Instability-Associated mRNAs (GI-mRNAs) in LUAD

**Figure 5:**
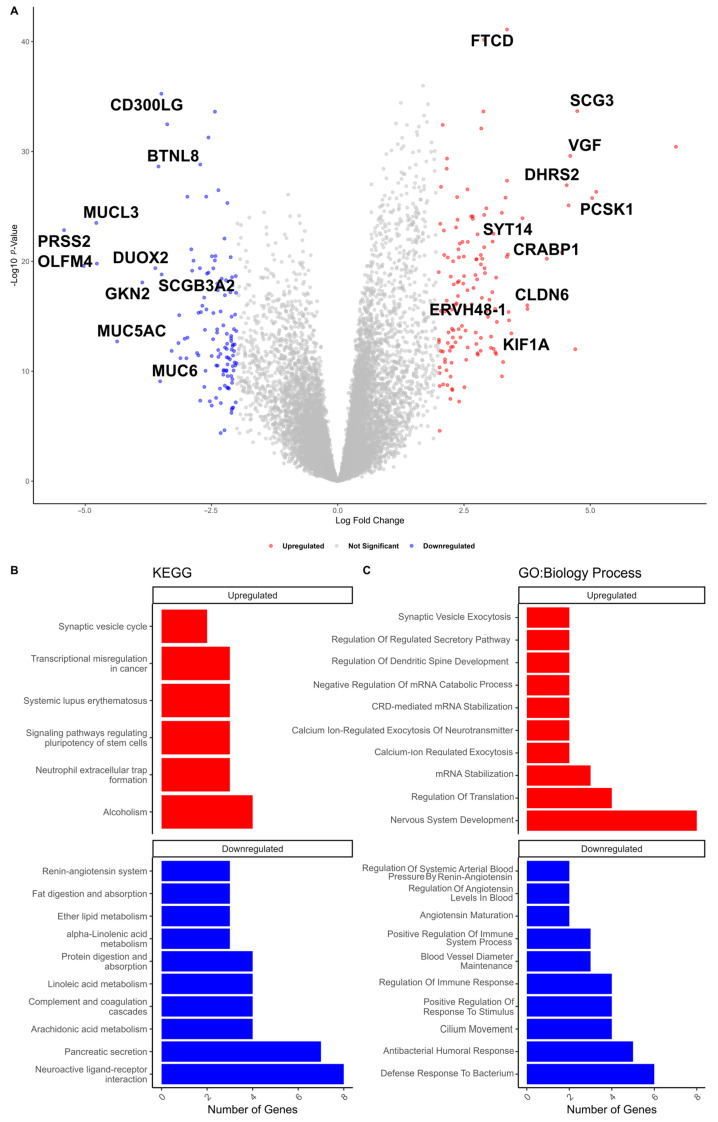
(**A**) Volcano plot showing upregulated and downregulated genes. Red points represent upregulated mRNAs, and blue points indicate downregulated mRNAs in samples with high HRD scores compared to samples with low HRD scores, defined by a log2 fold change ≥ |2| and an FDR < 0.05. The top 10 upregulated and downregulated GI-associated mRNAs—those that are both differentially expressed and significantly correlated with HRD scores (Spearman correlation)—are labeled. Gray points represent genes that are not differentially expressed. (**B**) Bar graph displaying the top 10 enriched pathways based on the *p*-value from the Kyoto Encyclopedia of Genes and Genomes (KEGG) pathway enrichment analysis for GI-mRNAs. (**C**) Bar graph displaying the top 10 enriched biological processes, based on the *p*-value, from Gene Ontology (GO) enrichment analysis for GI-mRNAs.

To identify GI-mRNAs in LUAD, we first compared mRNA expression between samples with high HRD scores and those with low HRD scores to identify differentially expressed mRNAs (DE-mRNAs). This analysis revealed 257 DE-mRNAs, with 123 upregulated genes and 134 downregulated genes (log2 FC ≥ |2| and FDR < 0.05). The complete list of DE-mRNAs is provided in Supplementary Table S7. After identifying the DE-mRNAs, we conducted a correlation analysis to assess the significance and nature of the association between HRD scores and DE-mRNA expression. We defined DE-mRNAs with a *p*-value < 0.05 and a Spearman coefficient ≥ |0.1| as GI-mRNAs. We identified a total of 200 GI-mRNAs (85 upregulated and 115 downregulated; Supplementary Table S8). The top 10 upregulated and downregulated GI-mRNAs are highlighted in Figure 5A.

The biological process and enrichment annotation analysis were performed on the GI-mRNAs with the same significant difference direction. The results indicated that the upregulated GI-mRNAs are primarily associated with pathways related to ’transcriptional misregulation in cancer’, while the downregulated GI-mRNAs are predominantly linked to metabolic processes (Figure 5B). Biological process analysis revealed that key processes include those previously implicated in the function of lncRNAs, such as the ’Regulation of Immune Response’, ’CRD-mediated mRNA Stabilization’, and ’mRNA Stabilization’ (Figure 5C). The complete list of KEGG pathways and biological processes are shown in the Supplementary Tables S9 and S10.

### 3.4. Identifying Interactions Between GI-lncRNAs and GI-mRNAs

LncRNAs can bind directly to mRNAs or form complexes with RNA-binding proteins, stabilizing mRNAs to promote their translation or marking them for degradation. Recent studies on lncRNA–mRNA interactions have demonstrated their regulatory roles in various biological processes [82,83,84], highlighting the utility of comprehensive lncRNA–mRNA interaction predictions in elucidating lncRNA functions [85].

**Figure 6:**
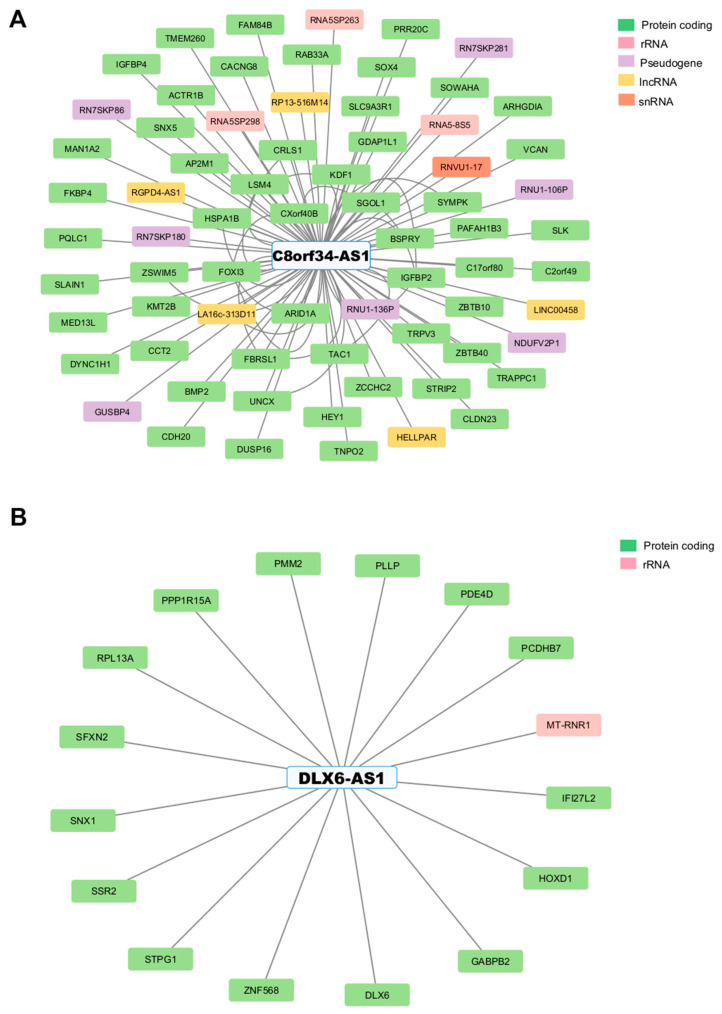
Interaction network depicting the associations involving the genomic instability-associated long non-coding RNAs (GI-lncRNAs) (**A**) *C8orf34-AS1* and (**B**) *DLX6-AS1*. The network was constructed using Cytoscape. rRNA: ribosomal RNA. lncRNA: long non-coding RNA. snRNA: small nuclear RNA.

To investigate the potential interactions between GI-lncRNAs and GI-mRNAs in LUAD, we utilized the RISE database, an RNA interactome database from sequencing experiments [57]. From this analysis, we identified 132 interactions involving GI-lncRNAs (Supplementary Table S11). Notably, 12 out of 30 GI-lncRNAs were found to interact with other protein-coding and non-coding RNA molecules. These GI-lncRNAs include *DLX6-AS1*, *C8orf34-AS1*, *LINC01224*, *LINC00664*, *LHFPL3-AS2*, *MIR2052HG*, *CALML3-AS1*, *SRGAP3-AS2*, *TRPM2-AS*, *SFTA1P*, *ATP13A4-AS1*, and *MGC27382*. The GI-lncRNAs *C8orf34-AS1* and *DLX6-AS1* exhibited the highest number of interactions among all identified GI-lncRNAs (Figure 6A,B). Specifically, *C8orf34-AS1* demonstrated a total of 70 interactions, including 54 with mRNAs (protein-coding genes) and 5 with other lncRNAs (Figure 6A).

**Figure 7:**
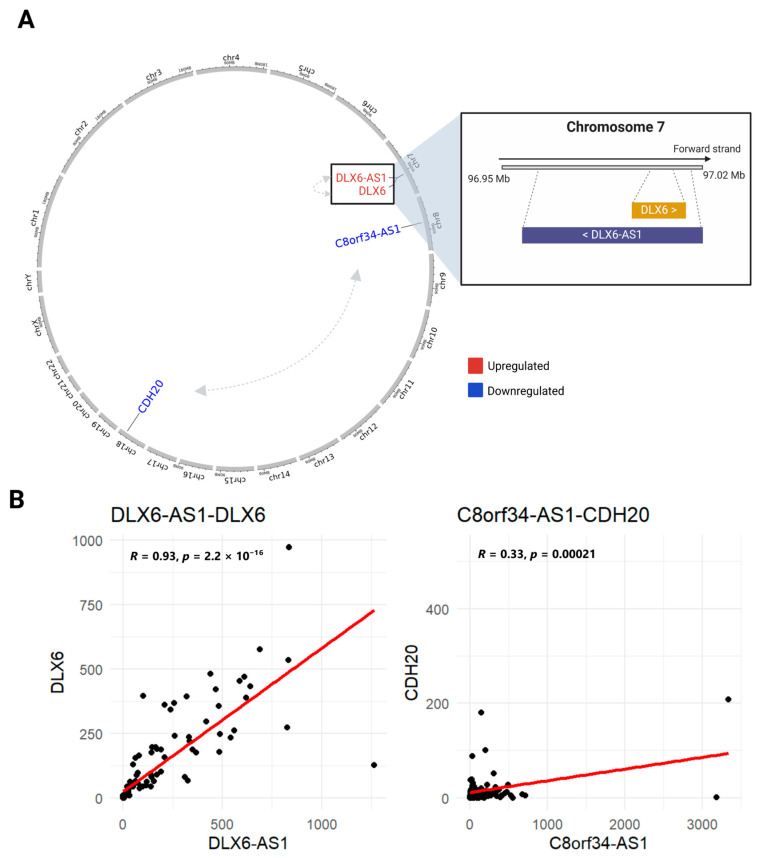
Genomic positions of GI-lncRNAs and GI-mRNAs with interactions identified in the RISE database. (**A**) The genomic positions of GI-lncRNAs and their corresponding GI-mRNAs are displayed. The figure was created using shinyCircos-V2.0 [86]. Grey arrows indicate interactions between GI-lncRNAs and GI-mRNAs. The zoomed-in box highlights chromosome 7, showing the overlap between the gene *DLX6* and its antisense, *DLX6-AS1*. (**B**) Spearman’s rank correlation comparing the expression levels of GI-lncRNAs and GI-mRNAs.

From the initial 132 interactions involving GI-lncRNAs, we filtered the dataset to include only interactions involving GI-mRNAs, identifying three significant interactions with *DLX6*, *FOXI3*, and *CDH20.* Specifically, *DLX6* was found to interact with *DLX6-AS1*, while *FOXI3* and *CDH20* were found to interact with *C8orf34-AS1*. In our cohort, we found a significant positive correlation between *DLX6-AS1* and *DLX6* (ρ = 0.93, *p* < 0.05), as well as between *C8orf34-AS1* and *CDH20* (ρ = 0.32) (Figure 7A,B).

The genomic localization of GI-lncRNAs and their associated GI-mRNAs suggests that these lncRNAs may have both cis- and trans-regulatory roles. For example, the lncRNA *DLX6-AS1* overlaps its corresponding protein-coding gene, *DLX6*, on chromosome 7q21.3, while *C8orf34-AS1* and *CDH20* localize to chromosomes 8 and 18, respectively.

### 3.5. Regulatory Mechanisms and Functional Roles of GI-lncRNAs in LUAD and Other Diseases

The interaction analysis in Section 3.4 offers insights into how GI-lncRNAs, such as *DLX6-AS1* and *C8orf34-AS1*, could regulate GI-mRNAs through cis- and trans-regulatory mechanisms, revealing a framework for their potential roles in LUAD biology. These specific interactions, such as the ones between *DLX6-AS1* and *DLX6* and between *C8orf34-AS1* and *CDH20*, highlight a direct molecular interplay that may influence tumor behavior in the context of genomic instability. Building upon these findings, Section 3.5 expands the focus to explore the broader regulatory mechanisms and functional roles of GI-lncRNAs in LUAD and other diseases by leveraging the LncTarD 2.0 database [56]. This comprehensive resource provides key lncRNA–target interactions, associated functions, and regulatory mechanisms in human diseases, encompassing over 70,000 interactions supported by manually curated experimental evidence. By integrating these two approaches, we develop a synergistic strategy to explore the dual aspects of GI-lncRNAs: their direct molecular interactions as well as their broader regulatory roles and implications across various disease contexts.

Among the 30 identified GI-lncRNAs, six upregulated lncRNAs (*DLX6-AS1, ELFN1-AS1, MIR2052HG, TRPM2-AS, LINC01224, and CALML3-AS1*) were found in the database, with functions predominantly linked to ceRNA activity, miRNA sponging, and transcriptional regulation across various cancer types, including NSCLC and LUAD.

For example, *DLX6-AS1*, the most highly expressed GI-lncRNA (logFC = 2.58), was implicated in multiple ceRNA interactions in cancer, acting as a miRNA sponge for several targets, such as *miR-16-5p*, *miR-505-3p*, and *miR-497-5p.* Notably, *miR-505* (logFC = 0.37) and miR-497 (logFC = −0.54) were also significantly dysregulated in GI-LUAD samples (*p* < 0.05; Supplementary Table S12). However, whether these dysregulations are directly mediated by *DLX6-AS1* or occur through indirect mechanisms in the context of GI in LUAD requires further investigation.

Tumor suppressor miRNAs such as *miR-497-5p* and *miR-140-3p* were also identified as targets of GI-lncRNA-mediated sponging. For instance, *TRPM2-AS* sponges *miR-140-3p*, a known tumor suppressor in NSCLC [87]. Similarly, *DLX6-AS1* accelerates cell proliferation by regulating the miR-497-5p/SNCG pathway in prostate cancer [88].

In addition to miRNA sponging, GI-lncRNAs are also involved in transcriptional regulation. *TRPM2-AS* interacts with the transcription factor *ELK1* in gastric cancer, while *LINC01224* interacts with *YY1* in colorectal cancer, both of which influence cell cycle progression and stress responses.

GI-lncRNAs have also been shown to interact with oncogenic targets. *DLX6-AS1* was found to regulate *PIK3CA* and *MTOR* in colorectal cancer. These genes are critical components of pathways governing cell survival, proliferation, and chromatin remodeling, such as the PI3K/AKT/mTOR signaling cascade. This illustrates the potential contribution of GI-lncRNAs to tumor progression in LUAD with genomic instability.

Table 3 summarizes the regulatory mechanisms and functional roles of GI-lncRNAs in human diseases, as identified in the LncTarD 2.0 database. The table highlights predicted functions such as ceRNA activity, miRNA sponging, and transcriptional regulation, providing a functional framework for these GI-lncRNAs in LUAD.

Interestingly, supporting our previous findings, there is evidence of an interaction between *DLX6-AS1* and *DLX6* in LUAD. However, the exact mechanism of this interaction remains unknown, and further studies are necessary to elucidate this.

### 3.6. GI-lncRNAs as Potential Prognostic Biomarkers in LUAD

The mechanistic interactions identified in Section 3.4, particularly those involving GI-lncRNAs such as *DLX6-AS1* and *C8orf34-AS1*, emphasize their potential regulatory roles in LUAD through both cis- and trans-regulatory mechanisms. These findings, coupled with evidence from the LncTarD 2.0 database in Section 3.5, establish a framework for understanding how GI-lncRNAs can contribute to LUAD-related processes, including miRNA sponging, ceRNA activity, and transcriptional regulation. The interplay between GI-lncRNAs and pivotal oncogenic pathways like PI3K/AKT/mTOR further highlights their potential as mediators of tumor progression.

Building on these mechanistic insights, in this section, we explore the clinical significance of GI-lncRNAs as prognostic biomarkers in LUAD.

**Figure 8:**
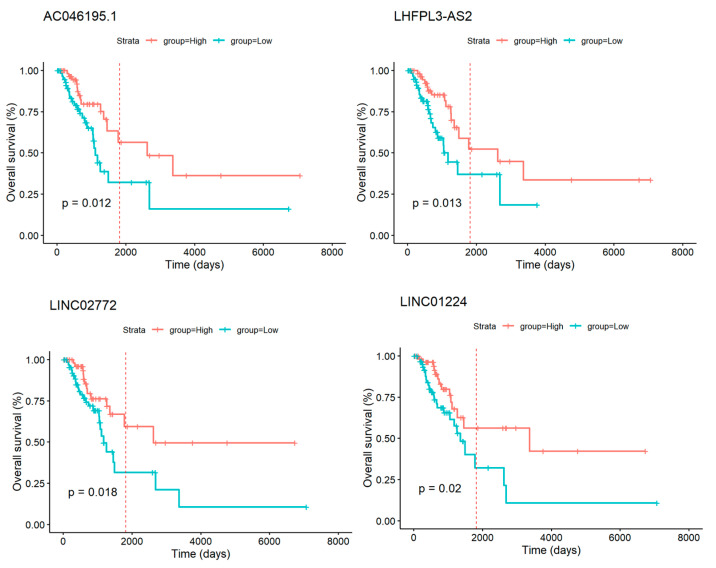
Kaplan–Meier survival curves for LUAD patients with high HRD scores, stratified by high and low expression levels of the target genes. Blue curves represent patients with low gene expression, while red curves denote those with high expression. The log-rank test was used to calculate the *p*-value, with *p* < 0.05 considered statistically significant. Dashed lines mark the 5-year survival time. The gene expression is normalized by z-scores, as described in the methodology section.

Our survival analysis revealed that specific GI-lncRNAs have significant prognostic value in LUAD patients stratified by HRD scores. For example, in the group with high HRD scores, low expression of GI-lncRNAs such as *AC046195.1*, *LHFPL3-AS2*, *LINC02772*, and *LINC01224* was associated with worse OS, suggesting their potential as biomarkers for poor outcomes in highly unstable genomic contexts (Table 4 and Figure 8).

We also explored if the GI-mRNAs (*DLX6* and *CDH20*) predicted to interact with GI-lncRNAs could be associated with OS, and curiously, we found that although *DLX6-AS1* and *C8orf34-AS1* were not associated with OS in samples with high HRD scores, high expression of GI-mRNA *DLX6* (logFC 3.33) was associated with worse OS in these patients (Supplementary Figure S2).

### 3.7. Association of GI-lncRNAs with Primary Therapy Outcomes

Given the association between GI-lncRNAs and survival in patients with high HRD scores, we further investigated their potential relevance in predicting therapy response in this subgroup. Our analysis revealed significant differences in the expression levels of *AC046195.1* (log2FC = −2.24) and *AC009244.1* (log2FC = −2.06), both of which were notably downregulated in patients with high HRD scores. Notably, lower expression levels of these lncRNAs were associated with progressive or stable disease, whereas patients with higher expression levels demonstrated a more favorable therapeutic response.

Interestingly, when we extended our analysis to patients with low HRD scores, no significant association was observed between the expression of these lncRNAs and primary therapy outcomes. This suggests that these GI-lncRNAs may function as specific predictive biomarkers for therapy response in patients with high HRD scores.

**Figure 9:**
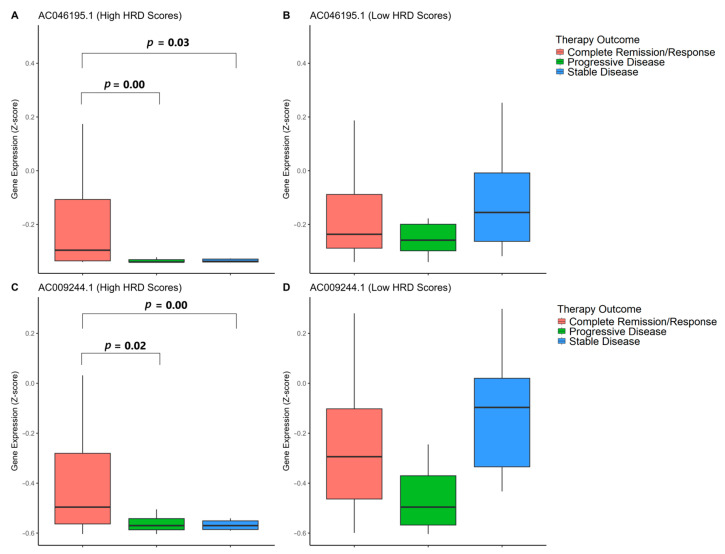
Fisher’s Exact Test results for the relationship between gene expression and therapy outcome success in samples with high and low HRD scores. (**A**) Expression of *AC046195.1* and therapy success in high HRD score patients. (**B**) Expression of *AC046195.1* and therapy success in low HRD score patients. (**C**) Expression of *AC009244.1* and therapy success in high HRD score patients. (**D**) Expression of *AC009244.1* and therapy success in low HRD score patients.

Figure 9 illustrates the expression levels of these GI-lncRNAs in patients stratified by HRD scores, while comprehensive data for all GI-lncRNAs are provided in Supplementary Tables S13 and S14.

### 3.8. Analysis of Immune Cell Infiltration in Tumors with High and Low HRD Scores 

Given that the tumor immune microenvironment can influence therapeutic response [89,90,91], we analyzed immune cell infiltration differences between patients with high and low HRD scores. Using the CIBERSORTx algorithm, we quantified immune cell infiltration in tumor tissues and identified significant differences (*p* < 0.05) between the two groups. In the low-HRD group, CD4 memory resting T cells comprised the largest proportion of immune cells (16.96%), whereas in the high-HRD group, M0 macrophages were the most abundant (16.04%) (Supplementary Table S15).

We observed significant differences in the infiltration levels of various immune cells, including plasma cells, T cells (CD4 memory resting, CD4 memory activated, and follicular helper), monocytes, macrophages (M0 and M1), dendritic cells (resting and activated), mast cells (resting), and neutrophils, between the groups (*p* < 0.05, Wilcoxon–Mann–Whitney test) (Supplementary Figures S3 and S4 and Supplementary Table S16).

**Figure 10:**
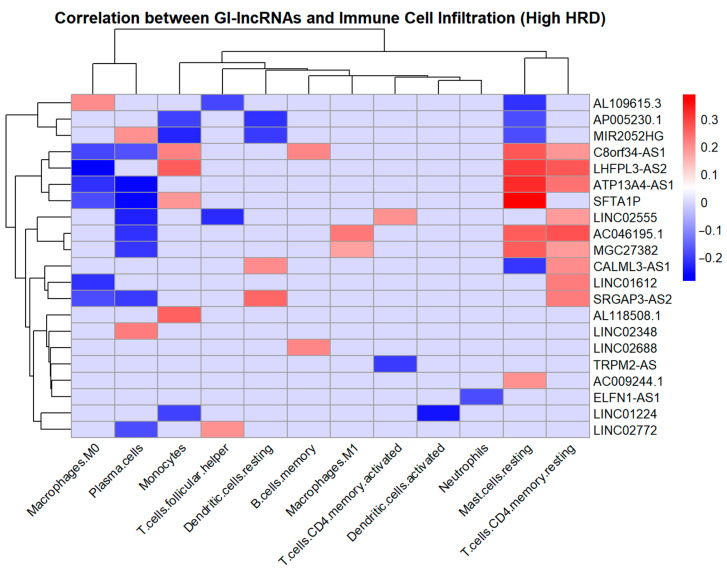
Heat map showing the correlation between GI-lncRNA expression and immune cell infiltration in the group with high HRD scores, using Spearman’s rank correlation. Red indicates a positive correlation, while blue indicates a negative correlation, with pale blue representing non-significant relationships (*p* ≥ 0.05).

To further investigate these findings, we examined the correlation between the infiltration of these immune cell types, which differed between the groups with high and low HRD scores, and the expression levels of GI-lncRNAs (Supplementary Table S17). We identified significant correlations between the expression of 21 out of 30 GI-lncRNAs and immune cell infiltration (Figure 10).

*C8orf34-AS1* was significantly associated with the greatest number of immune cell types, including B cells (memory), plasma cells, T cells (CD4 memory resting), monocytes, macrophages (M0), and mast cells (resting). Additionally, several GI-lncRNAs were found to be associated with the infiltration of T cells (CD4 memory resting), including *LHFPL3-AS2*, *LINC02555*, *C8orf34-AS1*, *AC046195.1*, *SRGAP3-AS2*, *MGC27382*, *ATP13A4-AS1*, *LINC01612*, and *CALML3-AS1*.

Interestingly, the GI-lncRNA *AC046195.1*, which is associated with primary therapy outcomes (Figure 9A), was correlated with the infiltration of plasma cells, T cells (CD4 memory resting), macrophages (M1), and mast cells (resting).

### 3.9. Exploring Drugs Targeting GI-lncRNAs

Our analysis identified 30 GI-lncRNAs with distinct expression patterns in LUAD, suggesting potential functional roles in tumor biology that warrant further investigation for both mechanistic insights and therapeutic development.

To investigate possible drug interactions with GI-lncRNAs, we utilized the ncRNADrug database, a comprehensive repository of experimentally validated and computationally predicted interactions between drugs and ncRNAs [79]. This approach enabled us to identify existing drugs that could interact with specific GI-lncRNAs, offering opportunities for drug repurposing in LUAD or for experimental research into their mechanistic roles in tumor progression.

Our findings show that most of the identified GI-lncRNAs are associated with one or more drugs (Supplementary Table S18). Table 5 details curated associations between select GI-lncRNAs and their corresponding drugs, including the cancer type in which these interactions were originally observed.

The lncRNA *DLX6-AS1*—which we identified as being associated with high HRD scores, with its predicted target protein-coding gene *DLX6* being linked to poorer prognosis—was found to interact with Panobinostat, an FDA-approved drug. This interaction could be leveraged in functional studies to investigate whether modulating *DLX6-AS1* affects genomic instability in LUAD, potentially revealing new therapeutic strategies.

Other notable GI-lncRNA-drug associations include Panobinostat with *KCNMB2-AS1* and *MIR9-1HG* (previously studied in nephroblastoma) and Dexamethasone with *ELFN1-AS1* (investigated in hepatocellular carcinoma). Many of these drugs are already approved for use in other cancers (for example, Oxaliplatin), providing a foundation for preclinical studies in LUAD, where their interactions with GI-lncRNAs could help uncover new mechanisms or therapeutic pathways.

## 4. Discussion

Lung cancers often exhibit asymptomatic progression, leading to diagnoses at advanced stages. Despite advancements in treatments, the five-year survival rate for lung cancer patients remains low [92], highlighting a critical need for new therapeutic strategies and diagnostic tools that can detect the disease earlier, where treatment is more effective. GI has been reported in various malignant cancers, including lung cancer [20,21,23,93], with increasing evidence suggesting that lncRNAs play crucial roles in regulating GI through diverse mechanisms [47]. Signatures of these lncRNAs hold promise as prognostic biomarkers in multiple cancer types. Previous studies have demonstrated that understanding genomic instability-related lncRNAs could help predict therapy responses and identify high-risk patients [94], and these lncRNAs may serve as novel therapeutic targets to address GI in cancers [47,50]. Recently, a signature comprising five genomic instability-associated lncRNAs was identified, demonstrating potential for predicting prognosis and serving as biomarkers in lung adenocarcinoma [50].

Numerous lncRNAs regulate DNA damage repair and genomic instability pathways [94,95,96]. In this study, we proposed identifying GI-associated lncRNAs in LUAD and their potential regulatory mechanisms and functional roles. We also explored the relationship between GI-lncRNAs and clinical outcomes, therapy responses, and immune infiltration. Additionally, we investigated potential drugs targeting GI- lncRNAs, aiming to identify existing drugs that could interact with them, offering opportunities for drug repositioning in LUAD or for experimental research into their mechanistic roles in tumor progression.

To classify TCGA samples, we used HRD scores, a well-established measure of GI [50,97]. HRD scores have been shown to be predictive of therapeutic outcomes in NSCLC patients undergoing neoadjuvant immunotherapy [98]. In ovarian cancer, individuals with HRD tumors experience a significant improvement in progression-free survival (PFS) with olaparib treatment [99], and the FDA has approved HRD as a companion diagnostic for the use of niraparib and olaparib in ovarian cancer therapy [100].

In this study, we explored the association between HRD scores and various clinicopathological characteristics, such as pathological stage, age, vital status, sex, race, and TNM classification. Although we did not observe a direct correlation between HRD scores and most of these features (Table 1), consistent with previous reports [25], we identified a notable association between HRD scores and age, suggesting that younger LUAD patients may exhibit higher levels of GI. This observation aligns with studies that have shown a negative association between age and GI in lung cancer, potentially due to tobacco exposure, which may lead to an earlier onset of mutation-dense lung cancers [101,102].

We also investigated the association between HRD scores and patient survival outcomes, specifically, OS and PFS. Patients with higher HRD scores exhibited lower OS and PFS, but the differences were not significant between the groups with high and low HRD scores. This lack of significance may seem counterintuitive given the established association between high HRD scores and lower PFS in eight cancer types (glioblastoma, esophageal cancer, thyroid carcinoma, kidney renal clear cell carcinoma, uterine corpus endometrial carcinoma, prostate adenocarcinoma, kidney renal papillary cell carcinoma, and adrenocortical carcinoma) [26]. However, it is important to note that the role of HRD in LUAD remains complex. A comprehensive pan-cancer analysis revealed that HRD scores varied significantly between patients with the same type of cancer, including lung cancers [37]. While HRD has been associated with favorable prognosis in some cancers, such as ovarian cancer and triple-negative breast cancer, its relationship with LUAD is not yet fully understood. A higher HRD score was also associated with worse PFS in multiple cancer types but not in lung squamous cell carcinoma (LUSC) and TCGA-LUAD cohorts [26]. Similar results were found by Feng et al., who did not observe a relationship between poorer OS and HRD score in the TCGA-LUAD cohort; however, in a different LUAD cohort, OS was significantly decreased in patients with high HRD scores [25].

Several factors, including the heterogeneity of the tumor microenvironment, the presence of co-mutations, and the intricate nature of genomic instability itself, could contribute to this lack of significant difference in survival outcomes [25]. Given the conflicting evidence, it would be accurate to say that the association between HRD scores and survival in LUAD is not consistently observed across all studies and may depend on various factors. Further studies with larger and more diverse cohorts and standardized methods for assessing genomic instability may help clarify this relationship. For now, it is important to note that GI impacts multiple aspects of tumor biology beyond survival, including tumor evolution and heterogeneity, immune evasion, and treatment resistance [103,104,105,106,107].

Although our findings suggest that HRD alone may not be a robust predictor of PFS or OS in LUAD, they highlight the potential of HRD as part of a composite biomarker. This emphasizes the need for an integrative approach that combines HRD with additional molecular features, such as specific lncRNAs, to improve patient stratification. Notably, combining HRD scores with *TP53* mutation status has been shown to enhance prognostic accuracy in LUAD [25].

In our study, LUAD patients with high HRD scores frequently harbored *TP53* mutations, while no significant differences were observed in the mutational status of other key driver genes. These findings are consistent with previous reports [25]. *TP53* mutations are strongly associated with genomic instability, including CIN, oncogene amplification, and tumor suppressor gene deletions [108]. *TP53* mutation-driven genomic instability is known to contribute to chemoresistance and recurrence, underscoring its broader implications in tumor progression [109].

Next, by comparing patients with high and low HRD scores, we identified 30 lncRNAs and 200 mRNAs associated with high genomic instability. Although numerous studies have focused on the individual roles of lncRNAs in a genomic instability context [50,110,111], a growing body of research has begun to investigate the combined influence of GI-associated lncRNAs and mRNAs [112,113,114]. In this study, we extended this approach by examining both GI-lncRNAs and GI-mRNAs, aiming to uncover potential interactions and regulatory mechanisms. This dual investigation enabled a more comprehensive understanding of how these molecular players collectively contribute to the GI phenotype in LUAD, potentially unveiling novel targets for therapeutic intervention and biomarkers for patient stratification. We identified three lncRNAs—*FAM83A-AS1*, *MIR193BHG*, and *LINC01116*—that have been previously reported in the literature as being associated with genomic instability in LUAD [42]. Although these lncRNAs did not meet our criterion of log2 fold change ≥ |2|, they all had an FDR < 0.05, indicating their potential relevance.

*DLX6-AS1* was the most upregulated GI-lncRNA (log2FC = 2.58). Recently, research groups have explored the expression of *DLX6-AS1* in human tissues and its clinical value in various cancers. All studies consistently concluded that *DLX6-AS1* is upregulated in patients with cancer (reviewed by Xue et al., 2020 [115]). Its oncogenic role has been extensively demonstrated in various cancer models, where it promotes an aggressive phenotype in cancer cell lines. Studies have shown that *DLX6-AS1* contributes to tumor progression by enhancing cell proliferation, migration, invasion, and epithelial–mesenchymal transition (EMT) while simultaneously suppressing apoptosis in cancer cells (reviewed by Ghafouri-Fard et al., 2022 [116]). In NSCLC, *DLX6-AS1* knockdown has been reported to suppress tumorigenesis and inhibit cancer progression [117]. Similarly, in pancreatic cancer (PC), the overexpression of *DLX6-AS1* was shown to drive tumor growth, as evidenced by increased tumor volume and weight, as well as a higher number of liver and lung metastatic foci [118]. Conversely, the knockdown of *DLX6-AS1* led to significant reductions in these oncogenic features, highlighting its potential as a therapeutic target. The overexpression of *DLX6-AS1* was also detected in lung cancer tissues and patients’ serum and exosomes and was positively correlated with a poor prognosis [119].

We found that GI-associated lncRNAs are distributed across the genome. This observation aligns with previous studies demonstrating the widespread nature of GI-related lncRNAs across chromosomal loci [47,50,120]. For instance, research on colon cancer identified a range of GI-associated lncRNAs and mRNAs spread throughout the genome [120]. Functional studies of lncRNA–mRNA co-expression networks highlight their varied influence in essential processes like immune responses, cell proliferation, and DNA damage repair [120].

One significant mechanism by which lncRNAs regulate gene expression is through post-transcriptional regulation [121,122,123], where they stabilize or destabilize messenger RNAs (mRNAs) via complementary base pairing, affecting mRNA degradation or translation. One class of lncRNAs involved in post-transcriptional regulation are known as competing endogenous RNAs (ceRNAs) or sponges [121,124]. LncRNAs can act as ceRNAs by two different mechanisms. On one hand, they are able to sequester miRNAs, avoiding their binding to target mRNAs, and on the other hand, they can directly interact with target mRNA transcripts to block miRNA binding sites [121,122,123]. Most lncRNAs that block miRNA activity to enhance mRNA stability are transcribed from the opposite DNA strand to their paired (sometimes complementary) sense protein-coding genes and are known as natural antisense transcripts (NATs) [125]. However, there are some examples of lncRNAs that can exert trans-regulatory actions, meaning they regulate genes located on different chromosomes or distant regions of the same chromosome, for example, *LincRNA-p21*, which recognizes mRNA targets by base pairing and regulates their translation [124,126]. Recent studies have highlighted the importance of lncRNA–mRNA interactions in regulating gene expression and cellular functions, underscoring the utility of computational and experimental approaches to predict and elucidate these interactions [82,83,84,85].

In this study, we explored potential interactions between GI-lncRNAs and GI-mRNAs in LUAD using the RISE database, which integrates RNA interactome data derived from sequencing experiments. Our analysis revealed notable interactions between *C8orf34-AS1* and *CDH20* and between *DLX6-AS1* and *DLX6*. *DLX6-AS1* is an antisense transcript of *DLX6*, a type of lncRNA that constitutes approximately 40% of all lncRNAs [127,128,129]. Antisense lncRNAs typically regulate their overlapping sense protein-coding genes through various mechanisms, often acting in cis due to their close genomic proximity. These mechanisms can influence the expression of neighboring protein-coding genes in either a concordant or discordant manner [130,131]. In our analysis, both *DLX6-AS1* and *DLX6* were found to be upregulated in high-HRD samples, suggesting a potential concordant regulatory relationship. Previous reports demonstrate that the expression level of *DLX6-AS1* in LUAD tissues is significantly higher compared to paired adjacent normal lung tissues. Furthermore, the downregulation of *DLX6-AS1* has been shown to decrease both *DLX6* mRNA and protein levels, providing further evidence of the regulatory interplay between these transcripts and highlighting their potential role in LUAD pathogenesis [132]. Additionally, using the LncTarD 2.0 database, a source that offers key lncRNA–target interactions, their associated functions, and regulatory mechanisms in human diseases, we found evidence of an interaction between *DLX6-AS1* and *DLX6* in LUAD. However, the exact molecular mechanisms underlying this interaction remain unclear, and further studies are needed to fully elucidate the biological significance and potential therapeutic implications of this regulatory relationship.

We also investigated the potential association between GI-lncRNAs and GI-mRNAs predicted to interact with them, based on the RISE database and their association with OS. Although *DLX6-AS1* and *C8orf34-AS1* were not associated with OS in samples with high HRD scores, high expression of the GI-mRNA *DLX6* (logFC = 3.33) was linked to worse OS in these patients (Supplementary Figure S2). This observation underscores the importance of further investigating the role of GI-mRNAs and their interactions with lncRNAs in LUAD prognosis and progression. This area opens avenues for directly targeting lncRNAs to affect the expression of their protein-coding partners. Recent advances in lipid nanoparticle (LNP) delivery systems, similar to those used in COVID-19 vaccines, enhance the feasibility of RNA-based therapies aimed at lncRNAs [133]. Furthermore, techniques like gapmers can be utilized to upregulate the expression of sense partner genes linked to antisense lncRNAs [134,135]. These gene therapies could potentially be used to overcome GI in LUAD by targeting lncRNAs. Further experimental validation of these interactions could provide deeper insights into their functional significance and therapeutic potential.

We identified significant prognostic implications for specific GI-lncRNAs in LUAD patients when stratified by HRD scores. Notably, in the group with high HRD scores, low expression of GI-lncRNAs such as *AC046195.1*, *LHFPL3-AS2*, and *LINC02772* was associated with worse OS. This suggests that these lncRNAs could serve as biomarkers for poor outcomes in tumors with high levels of genomic instability; however, further validation is required in independent data. Interestingly, these associations were not observed in patients with low HRD scores, highlighting the distinct biological dynamics in LUAD subgroups defined by their HRD levels. We also investigated the relationship between GI-lncRNA expression and primary therapy outcomes. In patients with high HRD scores, a lower expression of *AC046195.1* and *AC009244.1* was linked to progressive or stable disease, whereas no significant differences were observed in patients with low HRD scores. These findings highlight the context-dependent prognostic significance of GI-lncRNAs, particularly in relation to the genomic instability captured by HRD scores.

GI involves a complex interplay between immune evasion and immune activation. While increased genomic heterogeneity among tumor cells can facilitate immune escape, high levels of genomic alterations may enhance immunogenicity by generating neoantigens recognized by the immune system, potentially leading to greater immune infiltration [104,136]. To investigate the relationship between GI-lncRNA expression and immune infiltration in LUAD, we analyzed immune cell populations across groups with high and low HRD scores. Significant variations in the infiltration of plasma cells, CD4+ T cells, monocytes, macrophages, dendritic cells, and neutrophils were observed. Notably, tumors with high HRD scores exhibited elevated levels of M0 and M1 macrophages compared to those with low HRD scores. This finding aligns with previous studies indicating that macrophages can contribute to DNA damage and GI in adjacent cells [137,138,139]. These macrophages release diffusible factors that induce stress signaling, DNA damage, and CIN in neighboring cells [140]. The observed associations between GI-lncRNA expression and immune cell infiltration point to a potential involvement of GI-lncRNAs in shaping the interaction between the tumor and the immune system in LUAD. However, further research is needed to unravel the mechanisms driving these relationships.

Finally, we explored drugs targeting GI-lncRNAs, which could offer therapeutic possibilities or serve as tools for studying GI-lncRNA mechanisms. The lncRNA *UCA1*, for example, has been associated with drug resistance in several cancers, including bladder and breast cancer. The overexpression of *UCA1* is linked to resistance to chemotherapeutic agents like cisplatin and gemcitabine [141,142], and targeting *UCA1* through knockdown strategies has been shown to restore drug sensitivity in resistant cancer cells [143]. The potential of targeting ncRNAs, particularly lncRNAs and miRNAs, to overcome cancer GI and therapy resistance has been widely explored [144]. Two therapeutic strategies have been proposed to restore radio- and chemotherapy response via the CIN pathway: accelerating CIN to generate less-fit karyotypes or inhibiting CIN to target a stable and genetically frozen cancer cell population [144]. It has been proposed that by overexpressing or inhibiting CIN-associated lncRNAs, CIN-induced resistance to therapy can be manipulated [144]. Our findings revealed several drugs targeting GI-lncRNAs, including those upregulated in samples with high HRD scores. One promising finding is Panobinostat, which targets *DLX6-AS1*, a highly promising GI-lncRNA that warrants further investigation as a potential candidate for future studies.

While we acknowledge that the therapeutic implications of targeting GI-lncRNAs are promising, we emphasize that our study is exploratory, aiming to identify potential biomarkers and investigate their functional roles. The feasibility and clinical application of these findings, particularly the targeting of *DLX6-AS1* with drugs like Panobinostat, require further experimental validation and clinical trials to confirm their therapeutic potential in LUAD.

## 5. Conclusions

In this study, we explored the role of GI-lncRNAs and GI-mRNAs in LUAD, focusing on their interactions and potential as prognostic biomarkers, particularly when combined with HRD scores. We also investigated their association with immune infiltration in high-GI samples and identified FDA-approved drugs targeting these GI-lncRNAs, which could be explored further in future therapeutic and functional studies. However, further research is necessary to delve deeper into the molecular mechanisms underlying GI-lncRNA interactions and their contribution to tumor progression in LUAD. Future studies should focus on validating these findings in larger cohorts, exploring the therapeutic potential of targeting GI-lncRNAs, and conducting functional assays to better understand their roles in LUAD. Additionally, experimental studies involving drug repositioning and RNA-based therapies could provide insights into overcoming therapy resistance in LUAD. The identification of novel lncRNA-targeted therapies offers exciting prospects for clinical applications in LUAD treatment.

## Figures and Tables

**Table 1:** Clinicopathological features of lung adenocarcinoma patients.

Characteristics		High HRD Scores (*n* = 120)	Low HRD Scores (*n* = 134)	*p*-Value *
Age	≤65	70 (58.33%)	48 (35.82%)	0.001
	>65	46 (38.33%)	79 (59.03%)	
	Unknown	4 (3.33%)	7 (5.22%)	
Sex	Female	57 (47.50%)	73 (54.48%)	0.287
	Male	63 (52.50%)	61 (45.52%)	
Race	Caucasian/ White	85 (70.8%)	102 (76.1%)	0.281
	Black/African American	20 (16.7%)	12 (9.0%)	
	Asian	2 (1.7%)	1 (0.7%)	
	Unknown	13 (10.8%)	19 (14.2%)	
Stage	Stages I–II	97 (80.83%)	103 (76.87%)	0.757
	Stages III–IV	21 (17.50%)	26 (19.40%)	
	Unknown	2 (1.67%)	5 (3.73%)	
T	T1–2	106 (88.33%)	113 (84.33%)	0.519
	T3–4	14 (11.67%)	21 (15.67%)	
N	N0	79 (65.83%)	87 (64.93%)	0.893
	N1–3	41 (34.17%)	41 (30.60%)	
	NX	0 (0.00%)	6 (4.48%)	
M	M0	77 (64.17%)	95 (70.90%)	0.950
	M1	4 (3.33%)	5 (3.73%)	
	MX	38 (31.67%)	32 (23.88%)	
	Unknown	1 (0.83%)	2 (1.49%)	

* Chi-Square test: The statistical analysis excludes the Unknown, MX, and NX sample categories. The primary tumor (T) category is used to define the size and extent of the main tumor, with values ranging from T0, indicating no evidence of the primary tumor, to T4, representing a larger or more invasive tumor. The regional lymph nodes (N) category indicates whether cancer has spread to nearby lymph nodes, with N0 indicating no spread and higher numbers (N1, N2, and N3), reflecting the increasing number and/or size of affected lymph nodes. The distant metastasis (M) category assesses whether cancer has spread to distant parts of the body, with M0 indicating no metastasis and M1 indicating that the cancer has spread. The NX and MX stages are used when the extent of cancer in lymph nodes or metastasis cannot be measured, respectively.

**Table 2:** Mutational status of key driver genes in lung adenocarcinoma patients stratified by HRD scores.

Genes		High HRD Scores (*n* = 120)	Low HRD Scores (*n* = 134)	*p*-Value *
*TP53*	Mutant	96 (80%)	18 (13.43%)	1 × 10^−4^
	Wildtype	22 (18.33%)	111 (82.09%)	
	Unknown	2 (1.67%)	5 (3.73%)	
*EGFR*	Mutant	10 (8.33%)	19 (14.18%)	0.184
	Wildtype	108 (90%)	110 (82.09%)	
	Unknown	2 (1.67%)	5 (3.73%)	
*KRAS*	Mutant	25 (20.83%)	38 (28.36%)	0.179
	Wildtype	93 (77.5%)	91 (67.91%)	
	Unknown	2 (1.67%)	5 (3.73%)	
*PIK3CA*	Mutant	5 (4.17%)	6 (4.48%)	1
	Wildtype	113 (94.17%)	123 (91.79%)	
	Unknown	2 (1.67%)	5 (3.73%)	
*CDKN2A*	Mutant	9 (7.50%)	5 (3.73%)	0.318
	Wildtype	109 (90.83%)	124 (92.54%)	
	Unknown	2 (1.67%)	5 (3.73%)	

* Chi-Square test: The statistical analysis excludes categories with unknown values.

**Table 3:** Functions involving GI-lncRNAs retrieved from the LncTarD 2.0 database.

lncRNA	Function	Target	miRNA	Disease
*DLX6-AS1*	ceRNA	*RUNX2*	*miR-505-3p*	Breast cancer
		*ARPP19*	*miR-16-5p*	Cervical cancer
		*CDK4*	*miR-124-3p*	Ewing’s sarcoma
		*PDK1*	*miR-4290*	Gastric cancer
		*POU2F1*	*miR-204-5p*	Gastric cancer
		*MMP2*	*miR-203a*	Hepatocellular carcinoma
		*WEE1*	*miR-424-5p*	Liver cancer
		*E2F1*	*miR-197-5p*	Malignant glioma
		*YAP1*	*miR-497-5p*	Neuroblastoma
		*PRR11*	*miR-144*	NSCLC
		*DLK1*	*miR-129-5p*	Osteosarcoma
		*GADD45A*	*miR-376c*	Pre-eclampsia
		*ERP44*	*miR-149-5p*	Pre-eclampsia
		*PTEN*	*miR-26a*	Renal cell carcinoma
	protein interaction	*NOTCH1*		Ovarian cancer
		*UPF1*		Thyroid cancer
	NA	*PIK3CA*		Colorectal cancer
		*AKT1*		Colorectal cancer
		*MTOR*		Colorectal cancer
		*DLX6*		Lung adenocarcinoma
	sponge	*miR-199a*		Cervical cancer
		*miR-497-5p*		Neuroblastoma/PDAC
		*miR-223*		Urinary bladder cancer
	transcript reg	*CADM1*		Hepatocellular carcinoma
*LINC01224*	ceRNA	*MYO6*	*miR-485-5p*	Colorectal cancer
		*AKT3*	*miR-485-5p*	Endometrial cancer
	sponge	*MIR2467*		Colorectal cancer/NSCLC
	transcript reg	*YY1*		Colorectal cancer
*ELFN1-AS1*	ceRNA	*MTA1*	*miR-1250*	Colorectal cancer
		*TRIM44*	*miR-4644*	Colorectal cancer
		*GFPT1*	*miR-183-3p*	Esophageal cancer
	NA	*Erk*		Colon adenocarcinoma
		*IRS1*		Colon adenocarcinoma
		*vimentin*		Colon adenocarcinoma
*TRPM2-AS*	ceRNA	*PYCR1*	*miR-140-3p*	Breast cancer
		*HMGA1*	*miR-195*	Gastric cancer
		*WEE1*	*miR-497*	Retinoblastoma
	protein interaction	*TAF15*		Colorectal cancer
	NA	*SHC1*		NSCLC
	sponge	*miR-140-3p*		Breast cancer
	transcript reg	*ELK1*		Gastric cancer
*CALML3-AS1*	ceRNA	*ZBTB2*	*miR-4316*	Urinary bladder cancer
*MIR2052HG*	histone mod and transcript reg	*LMTK3*		Breast cancer

Legend: ceRNA (competing endogenous RNA): a regulatory mechanism where lncRNAs act as molecular sponges, sequestering miRNAs to influence gene expression. NA (not available): interactions or functions that have not been annotated. NSCLC: non-small cell lung cancer. PDAC: pancreatic cancer. transcript reg: transcriptional regulation. histone mod: histone modification.

**Table 4:** Key results of the survival analysis for GI-lncRNAs in LUAD patients stratified by HRD scores. The table includes the log-rank test-derived p-values, hazard ratios (HRs), and log-fold change (LogFC) values for GI-lncRNAs that demonstrated significant prognostic value in the high-HRD group.

	Gene	HR (Hazard Ratio)	*p*-Value	LogFC
High HRD Scores	*AC046195.1*	2.255680678	0.014686109	−2.24
	*LHFPL3-AS2*	2.226216673	0.015577954	−2.52
	*LINC02772*	2.202470692	0.021184545	−2.09
	*LINC01224*	2.150362909	0.0234937	2.30

*p* < 0.05 based on the log-rank test.

**Table 5:** Drugs interacting with GI-lncRNAs using the ncRNADrug database.

GI-lncRNA	Drug	DrugBank Accession Number	Cancer Type	PMID	FDA
*KCNMB2-AS1*	Panobinostat	DB06603	Nephroblastoma	26176219	approved
*MIR9-1HG*	Panobinostat	DB06603	Nephroblastoma	26176219	approved
*MIR9-1HG*	Tetraarsenic Oxide	NA	Breast Cancer	33932728	NA
*ELFN1-AS1*	Dexamethasone	DB01234	Hepatocellular Carcinoma	29409992	approved
*DLX6-AS1*	Panobinostat	DB06603	Nephroblastoma	26176219	approved
*LHFPL3-AS2*	Oxaliplatin	DB00526	Gastric Cancer	29156779	approved
*LHFPL3-AS2*	Panobinostat	DB06603	Nephroblastoma	26176219	approved
*LINC01612*	Oxaliplatin	DB00526	Gastric Cancer	29156779	approved
*SFTA1P*	Panobinostat	DB06603	Nephroblastoma	26176219	approved
*LINC02688*	Panobinostat	DB06603	Nephroblastoma	26176219	approved

NA: not available.

## Data Availability

Transcriptome profiling for RNA and miRNA and clinicopathological features were retrieved from the Genomic Data Commons Data Portal (https://portal.gdc.cancer.gov/, accessed on 20 July 2024). Patient clinical information, including survival data and recurrence, was obtained from the Genomic Data Commons Data Portal (https://portal.gdc.cancer.gov/, accessed on 12 August 2024). Data on primary therapy outcome success were retrieved from the University of California Santa Cruz (UCSC) Xena browser (https://xenabrowser.net/, accessed on 12 August 2024).

## Supplementary Materials

The following supporting information can be downloaded at: https://www.mdpi.com/article/10.3390/cancers17060996/s1, Figure S1: Kaplan–Meier survival curves for (A) overall survival and (B) progression-free survival comparing the groups with high and low HRD scores; Figure S2: Kaplan–Meier survival curves for LUAD patients with high HRD scores, stratified by the target genes’ high and low expression levels; Figure S3: Bar graph illustrating the composition of infiltrating immune cells in groups with high and low HRD scores, summarized by the mean values for each group; Figure S4: Box plot showing immune cell infiltration levels between high and low HRD scores. Table S1: Homologous Recombination Deficiency (HRD) scores for LUAD-TCGA samples; Table S2: The clinicopathological characteristics of the patients included in this study; Table S3: The list of differentially expressed long non-coding RNAs (DE-lncRNAs); Table S4: Correlation between DE-lncRNA expression and HRD scores; Table S5: KEGG pathways enriched by genomic instability-associated lncRNAs (GI-lncRNAs); Table S6: Chromosomal locations of genomic instability-associated long non-coding RNAs (GI-lncRNAs); Table S7: The list of differentially expressed mRNAs (DE-mRNAs); Table S8: Correlation between DE-mRNA expression and HRD scores; Table S9: Kyoto Encyclopedia of Genes and Genomes (KEGG) pathway enrichment analysis for GI-mRNAs; Table S10: Gene Ontology (GO) enrichment analysis for GI-mRNAs; Table S11: RNA–RNA interactions involving GI-lncRNAs; Table S12: The list of differentially expressed miRNAs (DE-miRNAs); Table S13: Fisher’s Exact Test results for the relationship between gene expression and therapy outcome success in samples with high HRD scores; Table S14: Fisher’s Exact Test results for the relationship between gene expression and therapy outcome success in samples with low HRD scores; Table S15: Infiltrating immune cell composition in groups with high and low HRD scores, summarized by the mean values calculated for each group; Table S16: Wilcoxon–Mann–Whitney test results comparing immune cell infiltration levels between high and low HRD scores; Table S17: Correlation between the immune cell infiltration levels and expression levels of GI-lncRNAs (high HRD scores); and Table S18: Existing drugs that may interact with GI-lncRNAs, according to the ncRNADrug database.
