# Supplementary material for: Identification of Genomic Instability-Associated LncRNAs as Potential Therapeutic Targets in Lung Adenocarcinoma
Source: Cancers (Basel). 2025 Mar 15;17(6):996. doi: 10.3390/cancers17060996 (PMC11940503; doi:10.3390/cancers17060996)
Supplement: Supplementary file 1 [file cancers-17-00996-s001.zip › Supplementary Figures.pdf]

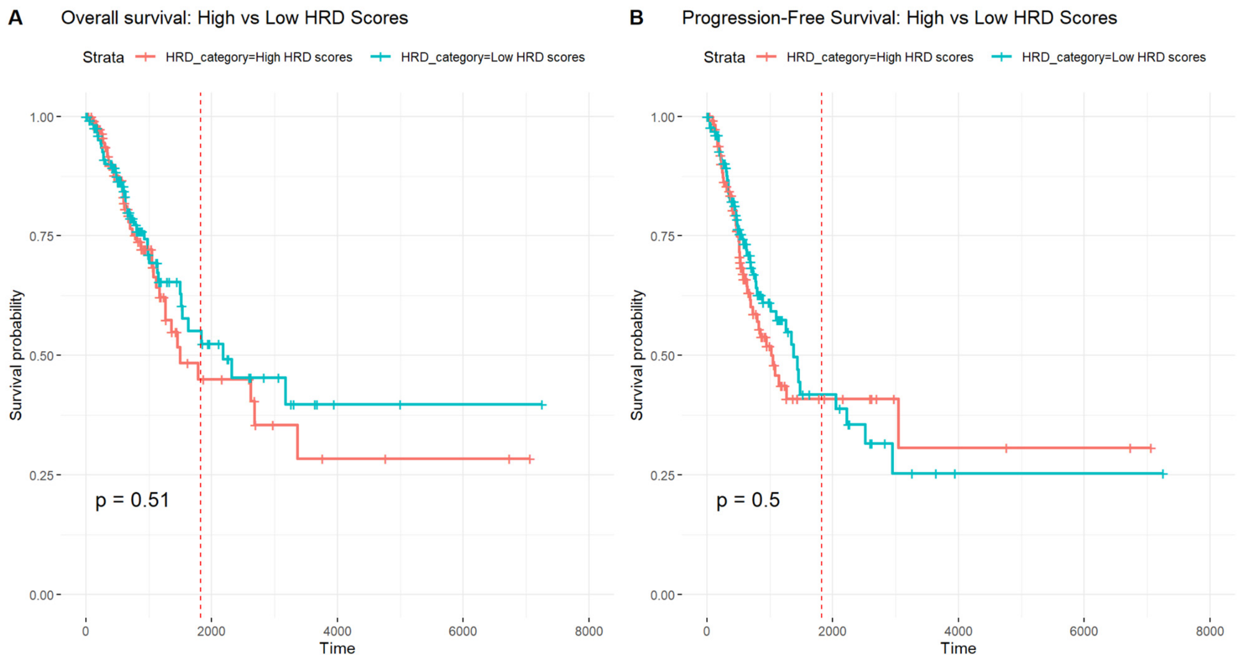

Figure S1. Kaplan-Meier survival curves for (**A**) overall survival and (**B**) progression-free survival comparing the groups with high and low HRD scores. Dashed lines mark the 5-year survival time.

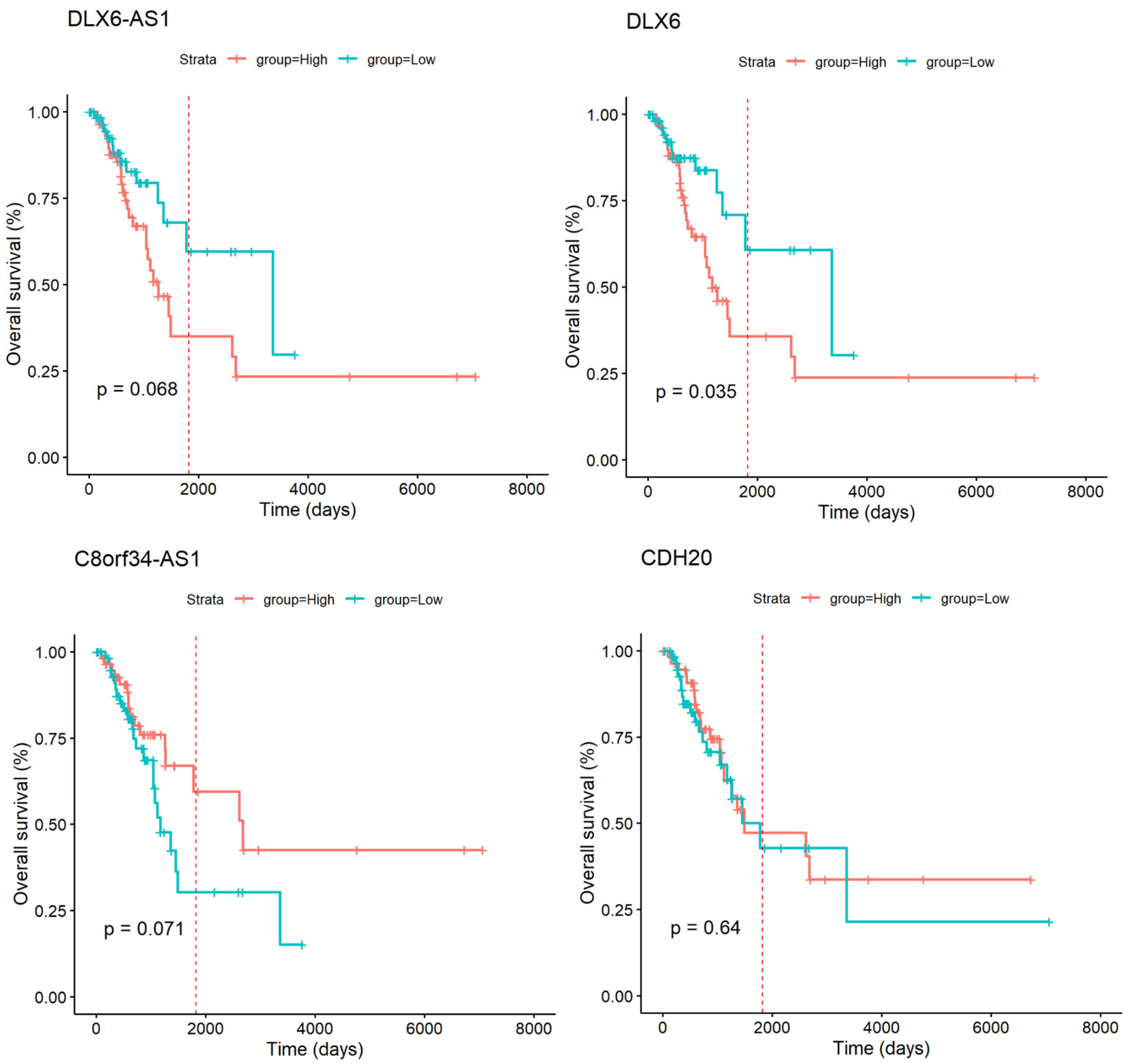

Figure S2. Kaplan-Meier survival curves for LUAD patients with high HRD scores, stratified by the target genes' high and low expression levels. Blue curves represent patients with low gene expression, while red curves denote those with high expression. The log-rank test calculated the *p*-value, with $p < 0.05$ considered statistically significant. Dashed lines mark the 5-year survival time. The gene expression is normalized by z-score as described in the methodology section.

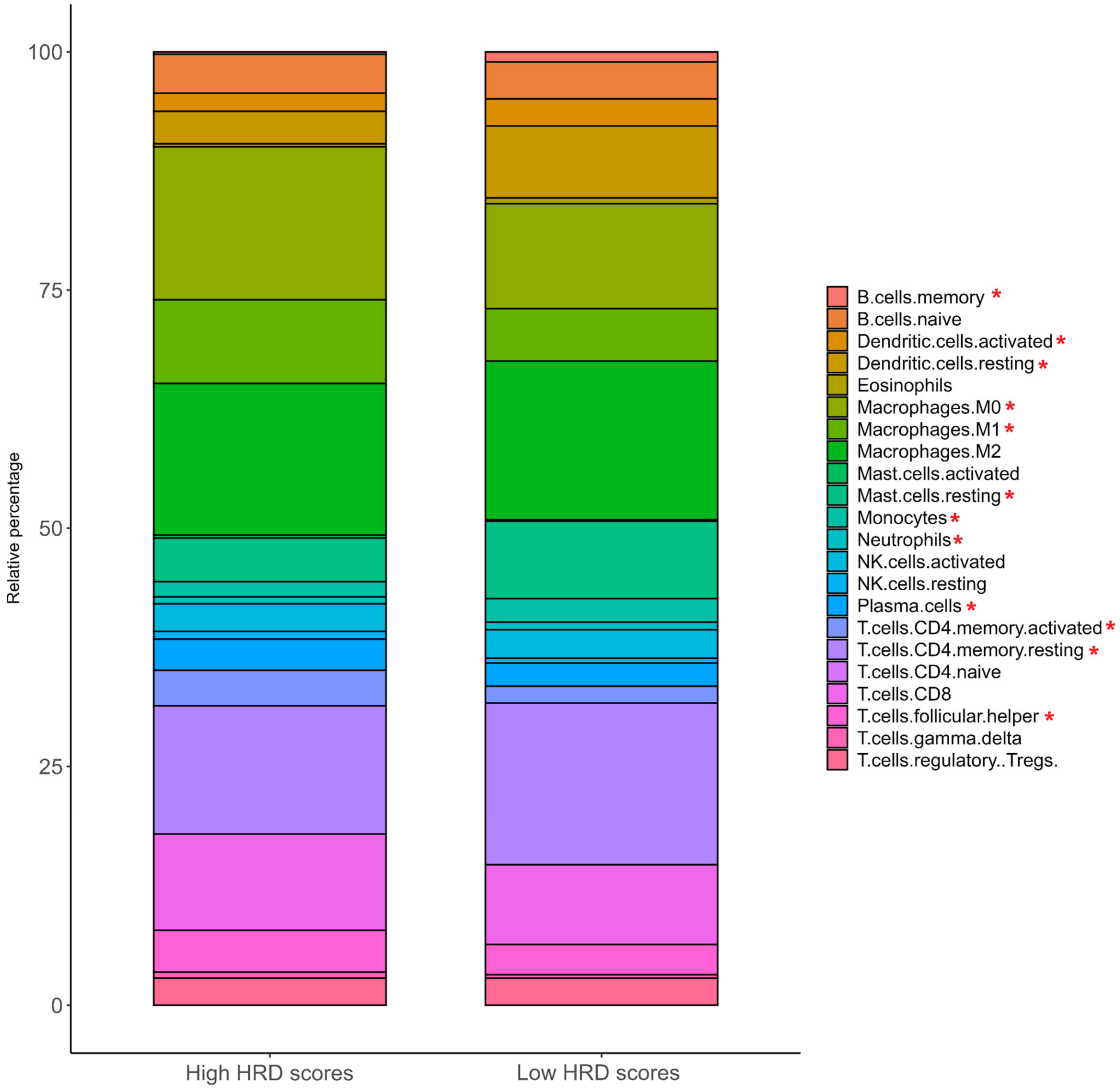

Figure S3. The bar graph illustrates the composition of infiltrating immune cells in groups with high and low HRD scores, summarized by the mean values for each group. An asterisk (*) indicates significant differences in immune cell infiltration levels between the high and low HRD score groups ($p < 0.05$, Wilcoxon–Mann–Whitney test).

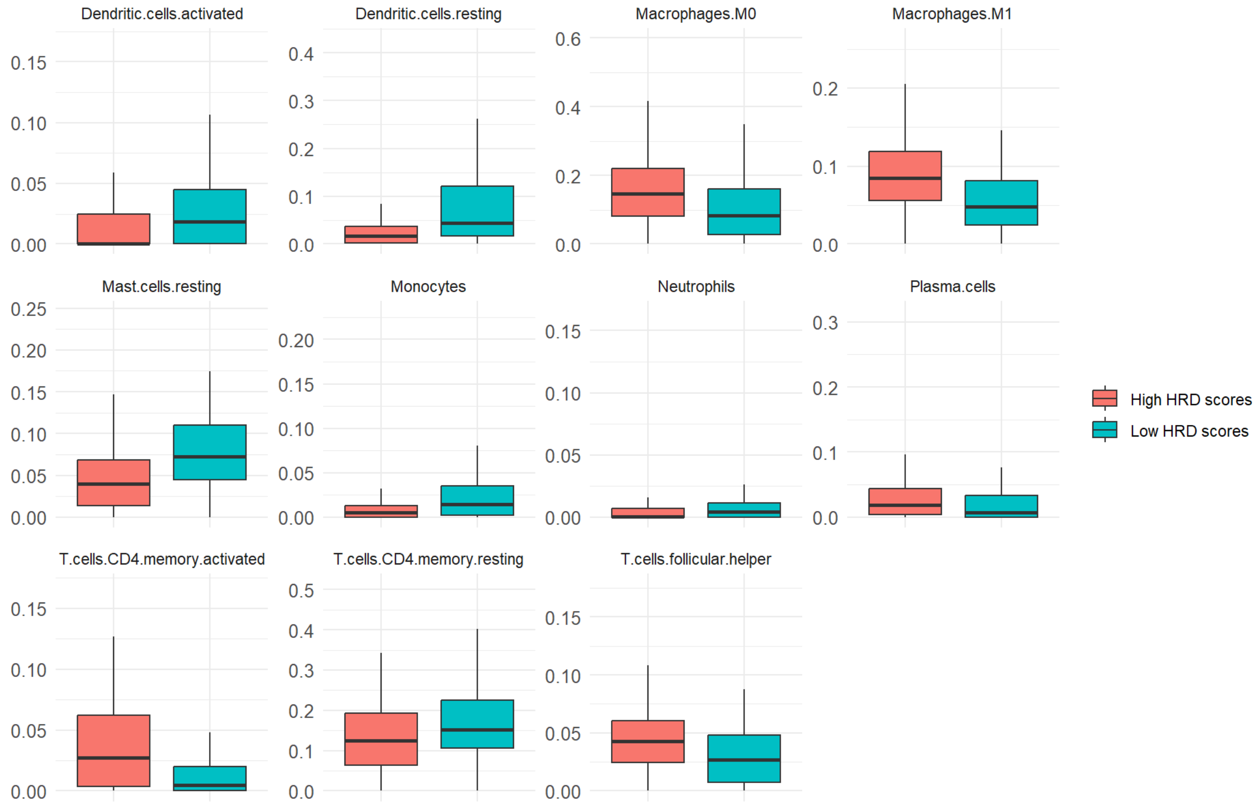

Figure S4. Box plot showing immune cell infiltration levels between high and low HRD scores for cells with significant differences ($p < 0.05$ Wilcoxon–Mann–Whitney test).
